# FXYD3 Promotes Tumor Progression by Binding With IRF7 to Regulate JAK2/STAT5 Signaling in Intrahepatic Cholangiocarcinoma

**DOI:** 10.1002/advs.202510782

**Published:** 2025-10-30

**Authors:** Yan Zhou, Xiaofeng Shen, Shuo Zhang, XiaoHong Pu, Zipeng Xu, Xiang Zhao, Wei Feng, Yuan Liang, Xingtao He, Aihua Yang, Yudong Qiu, Yihang Yuan, Chaobo Chen, Jun Chen

**Affiliations:** ^1^ Department of Pathology Nanjing Drum Tower Hospital The Affiliated Hospital of Nanjing University Medical School Nanjing 210000 China; ^2^ Department of Pancreatic surgery Nanjing Drum Tower Hospital The Affiliated Hospital of Nanjing University Medical School Nanjing 210000 China; ^3^ Department of general surgery Xishan people's hospital of Wuxi City Wuxi 214000 China; ^4^ Department of Pancreatic surgery Nanjing Drum Tower Hospital Clinical College of Nanjing Medical University Nanjing 210000 China; ^5^ School of Biological Science & Medical Engineering Southeast University Nanjing 210000 China; ^6^ Department of general surgery Nanjing Drum Tower Hospital Group Suqian Hospital Suqian 223800 China

**Keywords:** FXYD3, intrahepatic cholangiocarcinoma, IRF7, JAK2/STAT5 signaling, nanoparticles

## Abstract

Intrahepatic cholangiocarcinoma (ICC) has a poor prognosis, especially for inoperable patients. FXYD3, an FXYD‐domain‐containing regulator in the Na+/K+ ATPase family, is overexpressed in several common cancers. However, its role in ICC progression remains unclear. We integrated multiple ICC single‐cell transcriptome profiles from publicly available datasets and analyzed them using various bioinformatic methods, identifying FXYD3 as a candidate gene. In vitro and in vivo experiments demonstrated that FXYD3 expression was upregulated in ICC tumor tissues and associated with tumor progression and unfavorable prognosis. Subsequently, a combination of single‐cell sequencing, high‐resolution spatial transcriptome analysis, and a series of experimental assays demonstrated that FXYD3 directly interacts with IRF7 via its 60‐87aa domain, thereby initiating a positive feedback loop mediated by the cGAS/STING pathway. This loop is amplified by interferon type I and results in sustained activation of the JAK2/STAT5 signaling pathway, ultimately driving the malignant progression of ICC. The targeted FXYD3 nano‐delivery system (siFXYD3@PEP) exhibited significant antitumor efficacy in spontaneous and transplanted tumor models and markedly enhanced the sensitivity of ICC to standard gemcitabine and cisplatin chemotherapy. Our findings highlight the role of FXYD3 in cancer‐related inflammation and innate immune signaling, thereby providing a new paradigm for understanding the pathogenesis of ICC.

## Graphical abstract

FXYD3 binds IRF7 via its 60–87aa domain, triggering a cGAS/STING‐mediated positive feedback loop amplified by type I interferon. This sustains JAK2/STAT5 activation, accelerating ICC progression. Targeting FXYD3 with siFXYD3@PEP suppresses tumors and enhances chemotherapy sensitivity.

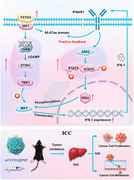

## 1 Introduction

In recent years, intrahepatic cholangiocarcinoma (ICC), the second most common malignant liver tumor, has witnessed an annual increase in morbidity and mortality.^[^ ^1^ ^]^ ICC is thought to stem from either bile duct epithelial cells or hepatic progenitor cells and is anatomically distinct from perihilar and distal cholangiocarcinomas.^[^ ^2^ ^]^ Owing to a shortage of effective screening strategies and the delayed manifestation of the disease, most patients present at an advanced stage.^[^ ^3^ ^]^ However, even when detected earlier, the tumor may be present in the liver or highly desmoplastic, which limits the sensitivity of cytological and pathological diagnostic methods.^[^ ^4^ ^]^ Consequently, only 20%–30% of patients are suitable for the surgical removal, which is the sole curative treatment. The standard of care for patients with unresectable disease or distant metastases currently includes first‐line therapy comprising gemcitabine and cisplatin, second‐line treatment involving the FOLFOX regimen, and adjuvant chemotherapy utilizing capecitabine.^[^ ^4^ ^]^ Although these systemic interventions have demonstrated efficacy in inhibiting tumor progression, the overall survival (OS) rate remains limited to approximately 1 year.^[^ ^5^, ^6^, ^7^ ^]^ Therefore, further investigation of the molecular mechanisms associated with ICC is imperative to improve patient survival and prognosis.

FXYD proteins, which consist of seven homologous single‐transmembrane proteins (FXYD1‐7), exhibit specific tissue expression patterns and act as a tissue‐specific regulator of Na, K‐ATPase.^[^ ^8^, ^9^ ^]^ These proteins interact with and modulate the kinetic properties of Na‐ and K‐ATPase to meet the requirements of a particular tissue, cell type, and physiological state without influencing other aspects.^[^ ^10^ ^]^ This allows the adaptation of the kinetic properties of active Na^+^ and K^+^ transport to meet the distinct needs of a variety of cells.^[^ ^11^, ^12^ ^]^ Particularly, FXYD3, also termed Mat‐8, is a tissue‐specific accessory subunit of Na, K‐ATPase^[^ ^13^, ^14^ ^]^ containing an invariant FXYD amino acid motif in its extracellular domain.^[^ ^15^ ^]^ Although FXYD3 is primarily expressed in the stomach and colon in normal tissues, it is overexpressed in certain diseases.^[^ ^16^ ^]^ For instance, FXYD3 expression is upregulated in multiple cancers, ranging from breast, pancreatic, colon, and prostate cancers to endometrial cancer,^[^ ^17^, ^18^, ^19^ ^]^ and contributes to the appearance and progression of tumors as well as the occurrence of chemotherapy resistance, which indicates poor prognosis.^[^ ^20^ ^]^ Recent reports have also shown that FXYD3 is highly expressed in the skin lesions of patients with psoriasis and promotes the development of psoriasis by regulating the IL‐17 signaling pathway.^[^ ^20^ ^]^ However, the role of FXYD3 in the development of ICC remains unclear.

IRF7 operates downstream of pattern recognition receptor (PRR) signaling and serves as a master regulator of type I interferon (IFN‐I) production. Upon phosphorylation and nuclear translocation, IRF7 promotes the transcription of IFN‐I genes, which bind to the interferon‐alpha receptor (IFNAR) on the cell surface, thereby activating the JAK/STAT signaling pathway. This cascade ultimately induces the expression of IFN‐stimulated genes (ISGs), establishing a positive feedback loop known as the IRF7‐IFN‐I axis.^[^ ^21^ ^]^

In this study, we integrated multiple ICC single‐cell transcriptome datasets and utilized a combination of bioinformatic approaches, which identified FXYD3 as a candidate gene. Subsequently, in vitro and in vivo experiments demonstrated that FXYD3 expression was significantly upregulated in ICC tumor tissues and cell lines and was closely associated with tumor progression and poor prognosis. The FXYD3‐mediated progression of ICC involves multiple biological processes, including migration, proliferation, invasion, metastasis, and lung metastasis. Therefore, we performed single‐cell sequencing integrated with high‐resolution spatial transcriptome analysis, complemented by a comprehensive series of in vitro and in vivo experiments, to elucidate the underlying mechanisms. Our findings demonstrated that FXYD3 directly interacted with IRF7 via its 60–87aa domain, thereby initiating a positive feedback loop mediated by the cGAS/STING pathway, and was amplified by IFN‐I. This loop results in sustained activation of the JAK2/STAT5 signaling pathway, which ultimately drives the malignant progression of ICC. Additionally, the targeted FXYD3 nano‐delivery system (siFXYD3@PEP) exhibited significant antitumor efficacy in spontaneous and transplanted tumor models and markedly enhanced the sensitivity of ICC to standard gemcitabine and cisplatin chemotherapy. Collectively, these findings underscore the critical role of FXYD3 in the development of ICC and highlight its potential as a therapeutic target.

## 2 Results

### 2.1 An Integrated Single‐Cell Transcriptomic Profiling of Intrahepatic Cholangiocarcinoma

**Figure 1:**
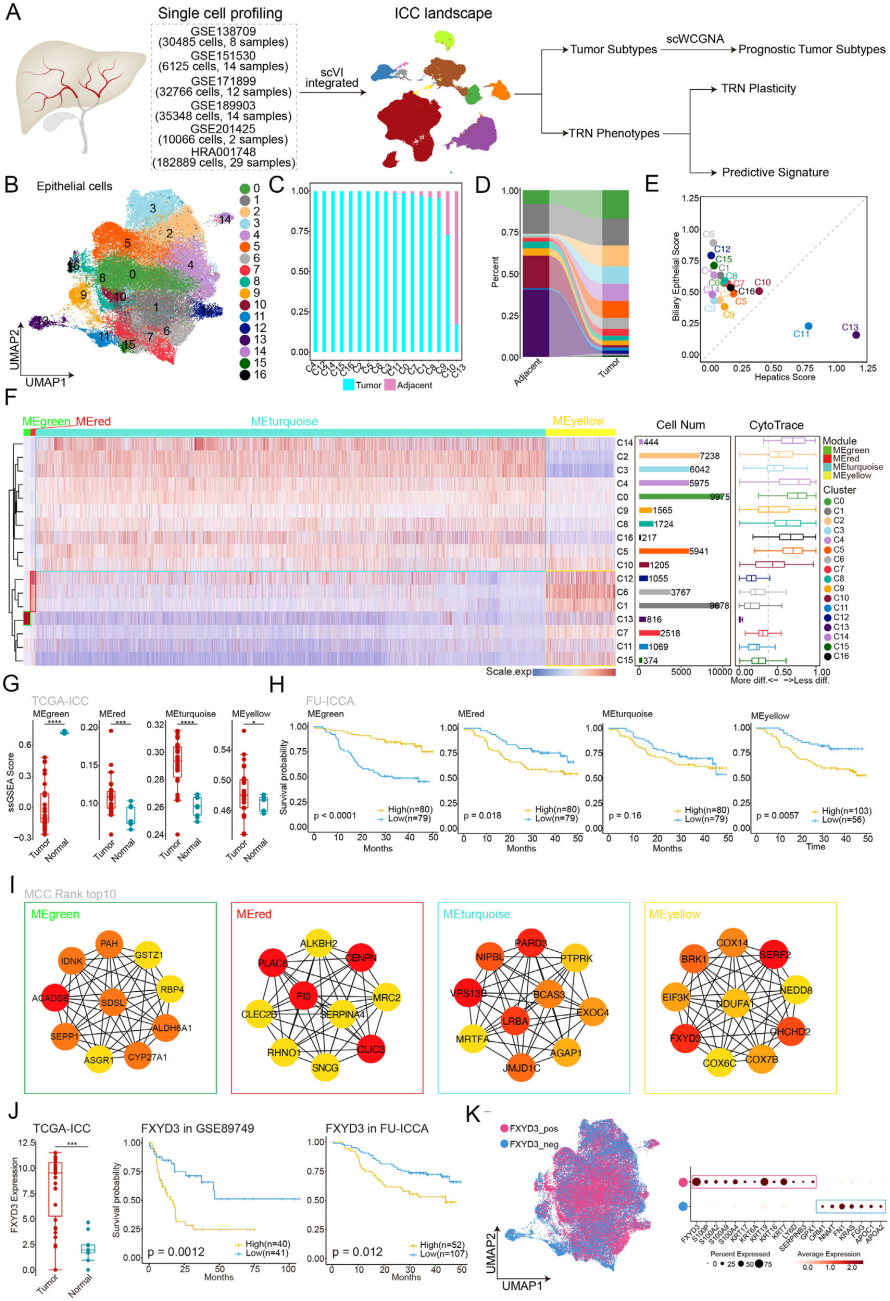
Integrated single‐cell transcriptomic profiling of intrahepatic cholangiocarcinoma. A) The flowchart for analysis and validation of integrated single‐cell sequencing data for intrahepatic cholangiocarcinoma. B) Uniform manifold approximation and projection (UMAP) plot showing the cell subtypes in the epithelial lineage. Dots refer to individual cells, and colors refer to different clusters. C) Box plots show the sample type fraction in each cluster. Bright blue represents the tumor samples, and dark pink represents the normal samples. D) A Sankey diagram showing cell cluster enrichment differences in tumor and normal samples. Colors represent different cell clusters. E. Scatter plot showing the clusters' hepatics scores (x‐axis) and biliary epithelial scores (*y*‐axis). Colors represent different cell clusters. F) Left: Heatmap showing the mean score of four gene expression modules identified by the scWGCNA algorithm. Middle: Bar plots showing the cell number of each cluster. Colors represent different cell clusters. Right: Box plots showing the differentiation score of each cluster calculated by the cytotrace algorithm. G) Box plots showing the scores of four module signatures between tumor (red) and normal (blue) samples in the TCGA‐ICC (Wilcoxon's rank‐sum test, *p* < 0.0001: ^****^, *p* < 0.001: ^***^, *p* < 0.01: **, *p* < 0.05: ^*^). H) Kaplan–Meier curves showing the overall survival values of four module scores in FU‐ICCA. I) Top 10 genes interaction regulatory networks based on the MCC algorithm in each module. Colors from yellow to red indicate the rank from bottom to top. J) Box plot showing the expression of FXYD3 between tumor (red) and normal (blue) samples in the TCGA‐ICC; Kaplan–Meier curves showing the overall survival values of FXYD3 expression in GSE89749 (middle) and FU‐ICCA (right). K) Uniform manifold approximation and projection (UMAP) plots showing the FXYD3 type based on the FXYD3 expression; dots represent individual cells, pink colors refer to the FXYD3_high group, and blue color represents the FXYD3_low group (left). Dot plot showing the differentially expressed features between the FXYD3_high and FXYD3_low groups (right).

We used the scVI algorithm to integrate the ICC single‐cell transcriptome profiles of 297679 high‐quality cells from six public datasets with 79 samples in batches (**Figure** **1**A). Differential expression analysis was then performed, which identified cell type‐specific markers, distinguished into 11 distinct cell types (Figures S1A–C, S2A, Supporting Information). We re‐clustered all epithelial cells into 17 distinct clusters to further investigate the heterogeneity of tumor cells and the molecular characteristics of ICC (Figure 1B). Based on the analysis of sample sources, C10 and C13 exhibited higher infiltration in peritumoral samples and lower malignancy than other clusters (Figure 1C). Furthermore, a detailed examination of cluster proportions in different sample types confirmed that C10 and C13 were relatively abundant in para‐cancerous tissues, whereas C2–C6 were more common in tumor tissues (Figure 1D). Thus, based on the previous findings, it can be inferred that C13 represents a normal hepatocyte population. Additionally, most of the tumor samples among the clusters with high biliary epithelial cell scores, such as C6, C12, and C15, exhibited a higher degree of malignancy compared to other clusters (Figure 1E). Subsequently, four molecular features (MEgreen, MEred, MEturquoise, and MEyellow) of tumor cells were obtained using the scWGCNA algorithm to identify the molecular characteristics of the tumor cells. Among them, MEyellow represented the specific high expression characteristics of clusters C12, C6, C1, C13, C17, and C15; clusters with high MEyellow expression had lower CytoTrace scores and exhibited lower dedifferentiation characteristics compared to the highly expressed MEturquoise‐related clusters (Figure 1F). Using the ssGSEA algorithm, the four functional modules of the tumors were successfully mapped onto the TCGA‐iCCA cohort. Additionally, survival analysis revealed that samples exhibiting high expression of MEred and MEyellow features had unfavorable prognoses, whereas those with elevated expression levels of MEgreen features showed promising prognostic outcomes (Figure 1G,H).

The feature network of single‐cell WGCNA was imported into Cytoscape to further target the key feature genes of each functional module, and the MCC algorithm was used to identify the top 10 crucial feature genes within each module. FXYD3 emerged as a pivotal ME‐yellow feature gene (Figure 1I). Subsequently, in an external dataset, FXYD3 was specifically overexpressed in tumor tissues in TCGA‐iCCA. Additionally, two independent survival datasets demonstrated that patients with high expression levels of FXYD3 had a poor prognosis compared to those exhibiting low FXYD3 expression (Figure 1J). All epithelial cells were categorized into two groups according to their FXYD3 expression levels in a single‐cell dataset: FXYD3‐positive and FXYD3‐negative epithelial cells. Thereafter, we detected an elevated expression of several tumor cell‐associated markers, including S100P, KRT17, KRT16, KRT7, and LY6D, as previously reported (Figure 1K).

### 2.2 FXYD3 is Upregulated in ICC and Predictive of Advanced Progression and Poor Prognosis

**Figure 2:**
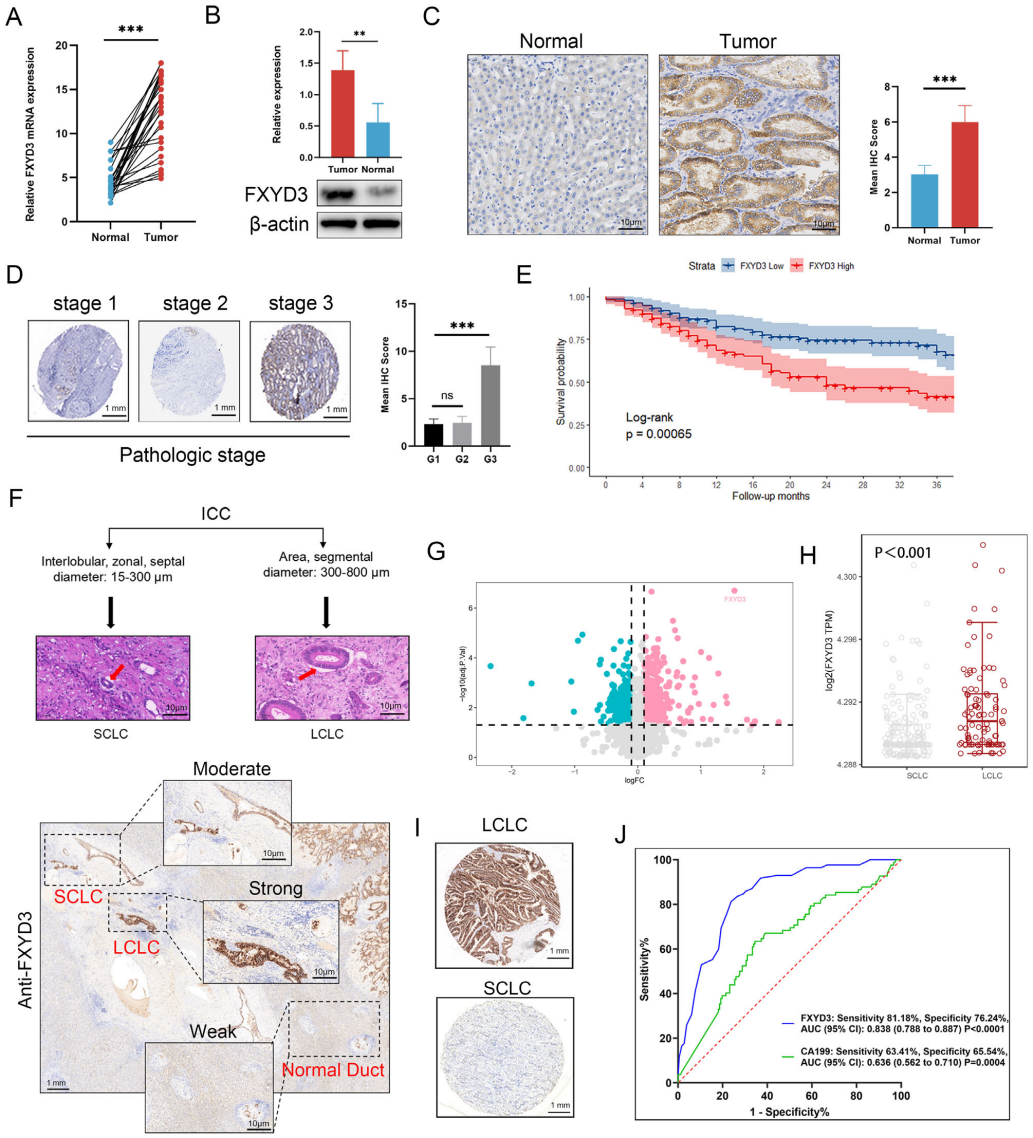
FXYD3 is upregulated in ICC, and predicts advanced progression and poor prognosis. A) Quantitative RT‐PCR analysis was performed to assess the expression of FXYD3 in ICC tissues and corresponding normal tissues (*n* = 50). B) Western blot was utilized to evaluate the expression level of FXYD3 in 10 randomly‐selected pairs of ICC and their paired adjacent tissues. C) IHC was performed to detect the expression of FXYD3 in tumor and adjacent normal samples, followed by quantification of IHC scores (*n* = 273). D) Tissue microarray staining was conducted to evaluate the expression pattern of FXYD3 under different pathological stages of ICC. E) High IHC scores for FXYD3 predict a poor survival rate (*P* = 0.00065). F) Schematic diagram illustrating the classification of ICC bile ducts (top); the global expression pattern of FXYD3 in the classification of ICC large and small bile ducts was evaluated using panoramic whole‐tissue sections (bottom). G, H) Differential expression patterns of FXYD3 were observed between the large bile duct subtype and small bile duct subtype within ICC cases (*n* = 273). I) Representative plots (*n* = 60) illustrate differences in FXYD3 expression between large and small bile ducts, as assessed by immunohistochemistry. J) Receiver operating characteristic curve (ROC) analysis demonstrating the diagnostic value of FXYD3 for distinguishing between small and large bile duct classification in ICC. Error bars show the mean ± SEM. ^*^ *p* < 0.05, ^**^ *p* < 0.01, and ^***^ *p* < 0.001. The *P* value was determined using two‐tailed unpaired Student's test or one‐way ANOVA.

We assessed the mRNA levels of FXYD3 in 25 pairs of ICC and adjacent nontumor samples using qRT‐PCR, and observed a substantial increase in FXYD3 mRNA levels in ICC samples compared to nontumor samples (**Figure** **2**A). In addition, western blot analysis of 10 randomly selected paired ICC tissue samples demonstrated significantly elevated FXYD3 levels in tumor tissues compared to non‐tumor tissues (Figure 2B). This finding was further validated by IHC staining of 273 clinical specimens with an FXYD3‐specific antibody (Figure 2C). Consistently, tissue microarray analysis revealed a significant increase in FXYD3 expression in ICC tissues, compared to nontumor tissues, which was positively correlated with pathological stage (Figure 2D). Survival analysis of the internal cohort (*n* = 273) showed that patients with ICC with high FXYD3 expression had poorer OS (Figure 2E).

Recent studies have stressed the importance and application of large and small bile duct classifications in ICC. We initially evaluated the expression levels of FXYD3 in various bile duct tissue types using pathological panoramic sections. The findings revealed that the expression of FXYD3 in large bile duct ICC was significantly higher than that in small bile duct ICC and normal bile duct tissue (Figure 2F). Subsequently, RNA‐Seq sequencing analysis of the relevant clinical samples (*n* = 273) was performed, along with immunohistochemical staining of the categorized clinical samples. The results indicated that FXYD3 expression was significantly associated with large duct‐type ICC (Figure 2G‐I). ROC analysis demonstrated that FXYD3 exhibited favorable diagnostic performance for large‐duct ICC, with a sensitivity of 81.18%, a specificity of 76.24%, and an AUC (95% CI) of 0.838 (0.788–0.887). Notably, the diagnostic efficacy of FXYD3 was superior to that of CA19‐9 [sensitivity, 63.41%; specificity, 65.54%; area under the curve, 0.636 (95% CI), (0.562–0.710); *p* < 0.05; *P* = 0.0004] (Figure 2J). These findings highlight the potential diagnostic value of FXYD3 in ICC classification.

In the subsequent clinicopathological analysis (**Table** **1**), the high FXYD3 group was significantly associated with large bile duct‐type ICC, poor differentiation (*P* = 0.001), poorer disease‐free survival (DFS, *P* = 0.004), and overall survival (*P* < 0.001) compared to the low FXYD3 group. Subsequently, univariate and multivariate Cox regression analyses were conducted to evaluate the potential of FXYD3 as an independent prognostic indicator of ICC. Univariate analysis revealed that high FXYD3 expression was significantly associated with OS (HR = 0.98; *P* < 0.001) (**Table** **2**). Variables with P < 0.05 were included in the multivariate Cox regression model, which indicated that FXYD3 (HR = 2.60; *P* = 0.004) was an independent predictor of worse OS (Table 2). Collectively, these findings indicate that FXYD3, which is highly expressed in ICC, can serve as a robust prognostic indicator of poor prognosis and disease progression.

**Table 1:** Comparative analysis of surgically treated patients with respect to FXYD3.

Variables	FXYD3 group		*P* value
	High (*n* = 139)	Low (*n* = 134)	
**Demographics**			
Gender, n (%)			0.060
Male	63 (45.3)	76 (56.7)	
Female	76 (54.7)	58 (43.3)	
Age (years)	62.6±8.7	60.3 ± 10.8	0.056
**Pathological examination**			
Single or multiple tumors, n (%)	26 (18.7)	22 (16.4)	0.620
Maximum tumor diameter (cm)	4.50 (3.20, 7.00)	5.25 (3.50, 7.50)	0.206
Microvascular invasion, n (%)	40 (30.1)	31 (23.8)	0.255
Histologic classification, n (%)			**<0.001**
Small duct	60 (44.8)	121 (91.7)	
Large duct	74 (55.2)	11 (8.3)	
Tumor grading, n (%)			**0.001**
G1	0 (0.0)	10 (7.5)	
G2	116 (86.6)	106 (79.7)	
G3	18 (13.4)	16 (12.0)	
G4	0 (0.0)	1 (0.8)	
T category, n (%)			0.084
T1	73 (54.5)	73 (56.2)	
T2	38 (28.4)	44 (33.8)	
T3	12 (9.0)	11 (8.5)	
T4	11 (8.2)	2 (1.5)	
N category, n (%)			0.042
Nx	19 (8.8)	35 (32.1)	
N0	45 (41.7)	43 (39.4)	
N1	43 (39.8)	31 (28.4)	
N2	1 (0.9)	0 (0.0)	
M category, n (%)			0.139
M0	130 (97.0)	130 (100.0)	
M1	4 (3.0)	0 (0.0)	
**Follow‐up Data**			
Recurrence‐free survival (months)	10.0 (6.0, 21.8)	19.5 (8.0, 35.3)	**0.004**
Overall survival (months)	11.0 (7.0, 26.0)	23.5 (11.0, 42.5)	**<0.001**

**Table 2:** Uni‐ and multivariate analysis of overall survival.

Variables	Univariate Analysis		Multivariate Analysis	
	HR (95% CI)	*P* Value	HR (95% CI)	*P* Value
FXYD3 (Low = 1)	1.98 (1.32‐2.97)	**<0.001**	2.60 (1.36, 4.96)	**0.004**
Gender (Male = 1)	1.20 (0.81‐1.78)	0.355		
Age	1.02 (1.00‐1.04)	0.057		
Single or multiple tumors	1.91 (1.19, 3.08)	**0.008**		
Maximum tumor diameter	1.11 (1.02, 1.20)	**0.012**	1.14 (1.04, 1.24)	**0.004**
Microvascular invasion	1.99 (1.32, 3.01)	**0.001**	1.99 (1.14, 3.47)	**0.016**
Histologic classification (Small duct = 1)	1.51 (1.00, 2.28)	**<0.05**		
Tumor grading (G1/G2 = 1)	2.64 (1.61, 4.33)	**<0.001**	3.91 (1.95, 7.86)	**<0.001**
T category (T1/T2 = 1)	2.18 (1.37, 3.49)	**0.001**		
N category (Nx/N0 = 1)	1.54 (0.98, 2.42)	0.062		
M category (M0 = 1)	6.91 (2.49, 19.19)	**<0.001**		

### 2.3 FXYD3 Promotes ICC Progression In Vitro and In Vivo

**Figure 3:**
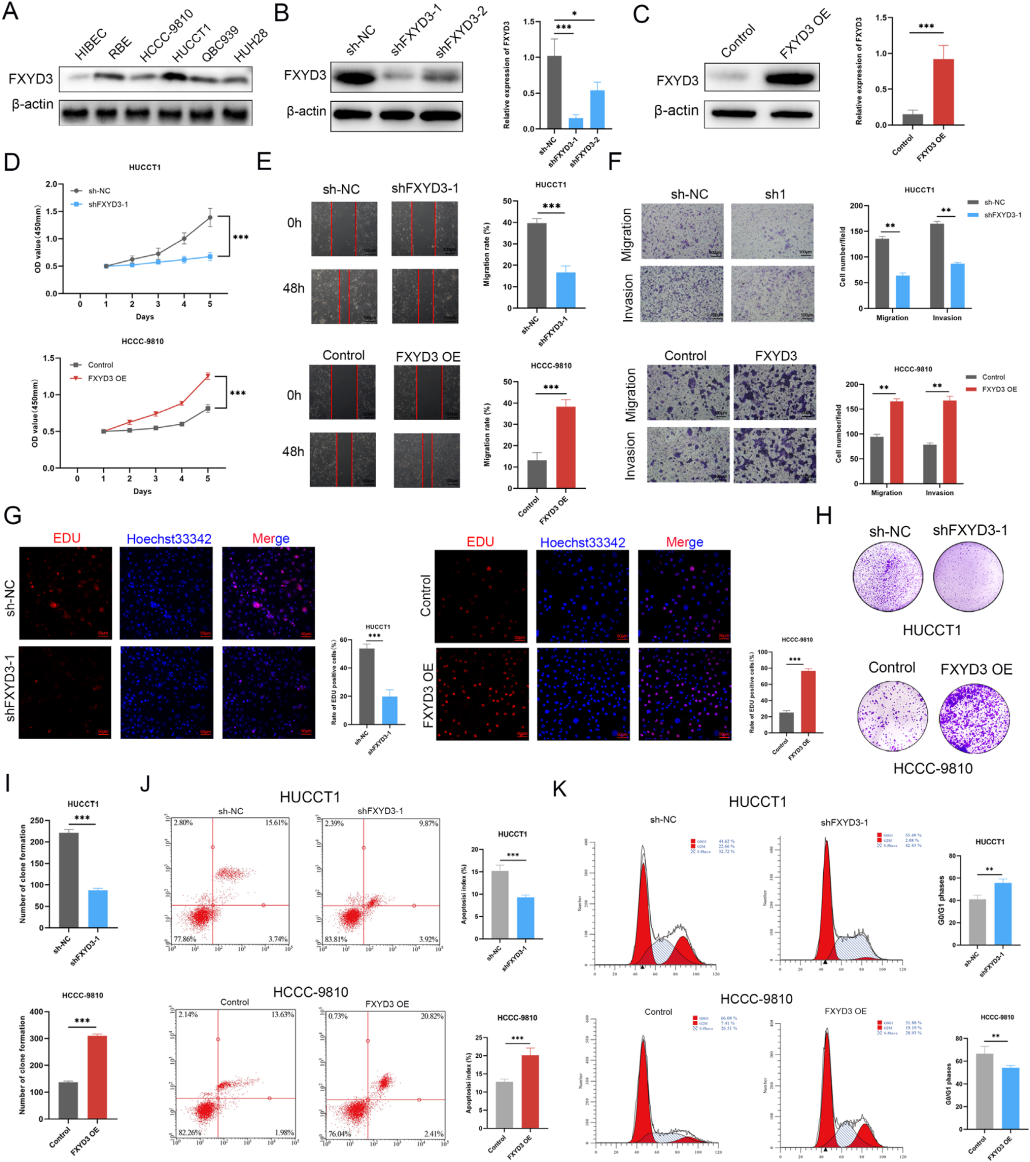
FXYD3 promotes ICC progression in vitro. A) Western blotting was used to determine the relative protein expression levels of FXYD3 in HIBEC cells and five cholangiocarcinoma cell lines. B, C) qRT‐PCR and western blot analysis were performed to assess the effectiveness of FXYD3 silencing and overexpression. D) CCK‐8 assay was performed to evaluate the proliferation ability of cells with FXYD3 knockdown or overexpression. E) A wound‐healing assay was used to determine the migration ability of cells with FXYD3 knockdown or overexpression. F) Transwell assay was employed to detect cell migration and invasion under conditions of FXYD3 knockdown or overexpression. G,H,I) EDU and colony formation assays were utilized to assess the proliferation ability of cells with FXYD3 knockdown or overexpression. J) Apoptosis of ICC cell lines with FXYD3 knockdown or overexpression analyzed by flow cytometry. K) Cell cycle analysis of ICC cell lines with FXYD3 knockdown or overexpression analyzed by flow cytometry. Error bars show the mean ± SEM. ^*^ *p* < 0.05, ^**^ *p* < 0.01, and ^***^ *p* < 0.001. The *P* value was determined using two‐tailed unpaired Student's test or one‐way ANOVA. Data are representative of three independent experiments.

We investigated the expression levels of FXYD3 in normal cholangiocytes (HIBEC) and cholangiocarcinoma cell lines (RBE, HCCC‐9810, HUCCT1, QBC939, and HUH28). The findings revealed markedly elevated surface expression of FXYD3 in cholangiocarcinoma cells compared to that in normal cholangiocytes (**Figure** **3**A). Therefore, HUCCT1 cells with high FXYD3 expression were selected for gene knockdown experiments, whereas HCCC‐9810 cells with low FXYD3 expression levels were selected for overexpression experiments. A lentivirus‐mediated FXYD3 short hairpin RNA vector was used to establish the corresponding cell lines featuring knockdown or overexpression, along with the control cell lines. The efficiency of knockdown or overexpression was determined using qRT‐PCR and western blotting (Figure 3B,C). Thereafter, we conducted CCK‐8, wound healing, Transwell, EdU, and clone formation assays using HCCC‐9810 cells overexpressing FXYD3 to better understand the functional role of FXYD3. The results of the CCK‐8 assay demonstrated that downregulation of FXYD3 expression significantly attenuated the proliferative capacity of HUCCT1 cells (Figure 3D **Up**). Subsequently, we performed wound healing assays to examine the effect of FXYD3 knockdown on tumor cell migration and invasion. As depicted in the figure, a notable reduction in HUCCT1 cell migration was observed following FXYD3 knockdown (Figure 3E **Up**). Moreover, the Transwell assay revealed that FXYD3 inhibition impeded cell migration and invasion (Figure 3F **Up**). Given the specificity and sensitivity of FXYD3, EdU incorporation was used to comprehensively assess its influence on cellular proliferation from several perspectives. The findings indicated that FXYD3 suppression led to significant inhibition of HUCCT1 cell proliferation (Figure 3G **left**). In addition, the clonal survival of HUCCT1 cells decreased notably following FXYD3 knockdown (Figure 3H **Up, I, Up**). To better understand the functional role of FXYD3, we conducted CCK‐8, wound healing, Transwell, EdU, and clone formation assay using HCCC‐9810 cells overexpressing FXYD3. As expected, overexpression of FXYD3 markedly enhanced proliferation as well as migration and invasion capabilities in HCCC‐9810 cells (Figure 3D **Down, E Down, F Down, G Right, H Down, I Down**). Apoptosis rate and cell cycle progression are critical factors in the occurrence and development of ICC. Our findings demonstrate that FXYD3 knockdown significantly promoted apoptosis in HUCCT1 cells, whereas FXYD3 overexpression in HCCC‐9810 cells markedly reduced apoptosis (Figure 3J). We examined whether FXYD3 influenced cell cycle distribution in ICC cells. Cell cycle analysis revealed that FXYD3 knockdown increased the proportion of HUCCT1 cells in the G0/G1 phase. Conversely, FXYD3 overexpression in HCCC‐9810 cells revealed a decreased proportion of cells in the G0/G1 phase (Figure 3K). Thereafter, key cell function experiments were performed independently and repeatedly using shFXYD3‐2 cells to avoid potential off‐target effects. Compared to the sh‐NC control, shFXYD3‐2 inhibited tumor cell proliferation, migration, and metastasis, while promoting apoptosis in vitro (Figure S3A–E, Supporting Information).

**Figure 4:**
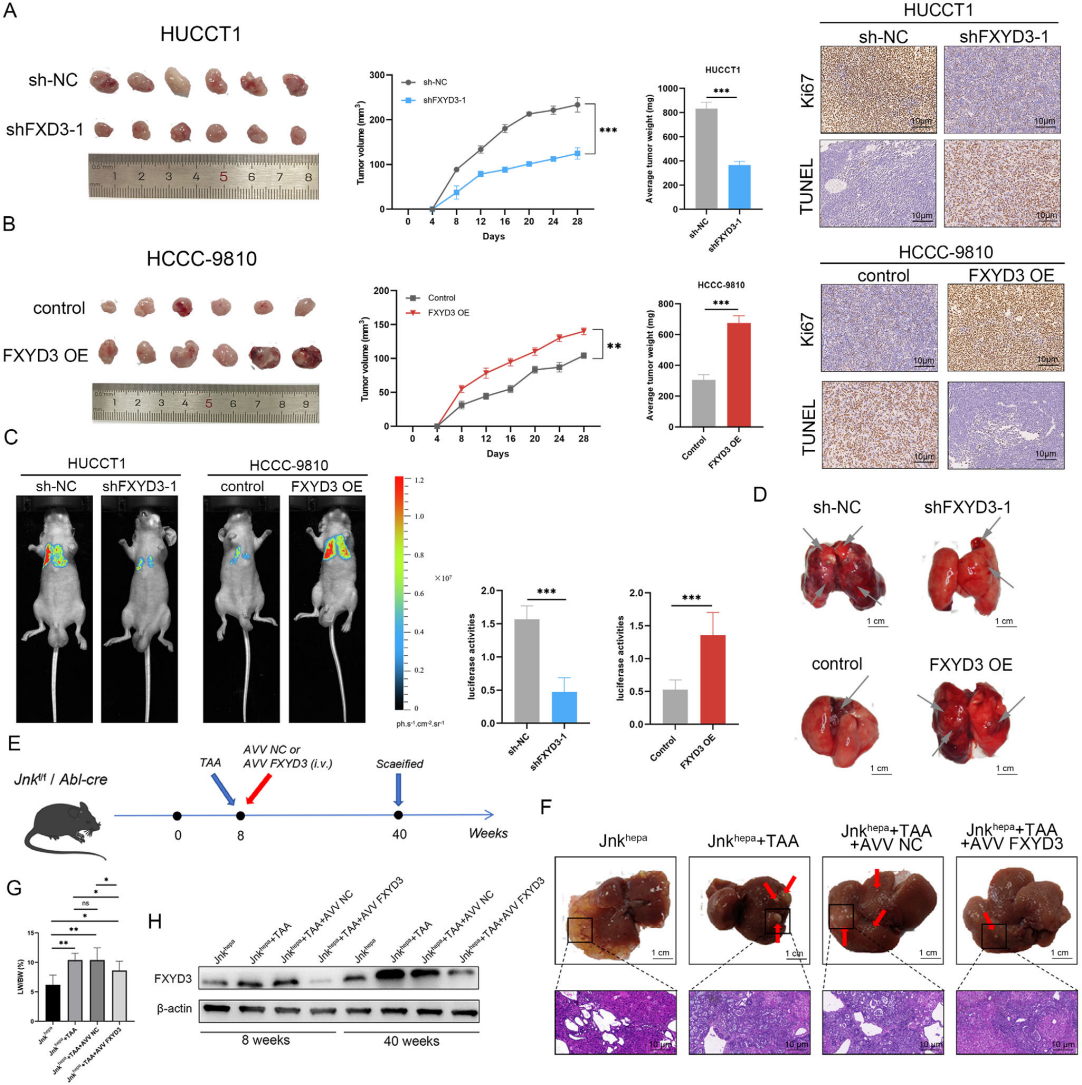
FXYD3 facilitates ICC progression in vivo. A,B) The representative images of ICC‐transplanted tumors in each group (*n* = 6) were captured, and the tumor volume growth curves as well as the tumor weights were analyzed and plotted. Immunohistochemical images depicting Ki‐67 and TUNEL expression in transplanted tumors from each group. C) Representative images of bioluminescence imaging in each group (*n* = 8). D) Representative gross picture of ICC lung metastasis. E) Spontaneous ICC model and FXYD3 knockdown model establishment. F) Representative images of liver tumors and HE staining in different groups (*n* = 8). G) Liver weight/body weight (LW/BW). H) Analysis of FXYD3 protein expression levels at various time points. Error bars show the mean ± SEM. ^*^ *p* < 0.05, ^**^ *p* < 0.01, and ^***^ *p* < 0.001. The *P* value was determined using two‐tailed unpaired Student's test or two‐way ANOVA. Data are representative of three independent experiments.

We examined the effect of FXYD3 on tumor growth in a mouse model of subcutaneously transplanted tumors to elucidate it's in vivo biological function. The results demonstrated that the injection of FXYD3 knockdown HUCCT cells resulted in slower tumor growth, compared to controls, as evidenced by reduced mean tumor weight and smaller tumor volume. Conversely, mice injected with FXYD3 overexpressing HCCC‐9810 cells exhibited accelerated tumor growth, with increased mean tumor size and weight, compared to controls. TUNEL staining and Ki‐67 staining further confirmed that FXYD3 promotes tumor formation in nude mice. Analysis of xenograft tumors revealed that the proliferative activity of shFXYD3‐1 tumors was significantly lower than that of sh‐NC control tumors, whereas the rate of apoptosis was higher. In contrast, the upregulation of FXYD3 in HCCC‐9810 xenografts resulted in enhanced proliferative activity and reduced apoptosis compared to sh‐NC control tumors (**Figure** **4**A,B). Additionally, FXYD3 knockdown or luciferase‐overexpressing ICC cells were injected into the tail vei ns of BALB/c nude mice to evaluate the biological role of FXYD3 in vivo. The findings indicated that FXYD3 downregulation in HUCCT1 cells was correlated with diminished lung metastasis, whereas FXYD3 overexpression in HCCC‐9810 cells was associated with a marked increase in lung metastasis (Figure 4C,D). Jnk^1/2^ (Jnk^∆hepa^) knockout mice were utilized to establish an ICC animal model with simultaneous administration of thioacetamide (TAA) to investigate the role of FXYD3 in a spontaneous ICC model. Subsequently, the impact of FXYD3 on ICC development was assessed by knocking down FXYD3 expression in mice through tail vein injection of adeno‐associated virus (AAV) shFXYD3 at 8 weeks (Figure 4E). FXYD3 knockdown resulted in a markedly reduced tumor burden, as evidenced by fewer tumors, smaller tumor volumes, and a decreased liver weight‐to‐body weight (LW/BW) ratio, compared to controls (Figure 4F,G). Furthermore, western blot analyses conducted at multiple time points (8 and 40 weeks) revealed a gradual increase in FXYD3 expression with ICC progression (Figure 4H). In conclusion, FXYD3 facilitated the malignant progression of ICC in vitro and in vivo.

### 2.4 FXYD3 Contributes to the Malignant Progression of ICC by Promoting JAK2/STAT5 Signaling

**Figure 5:**
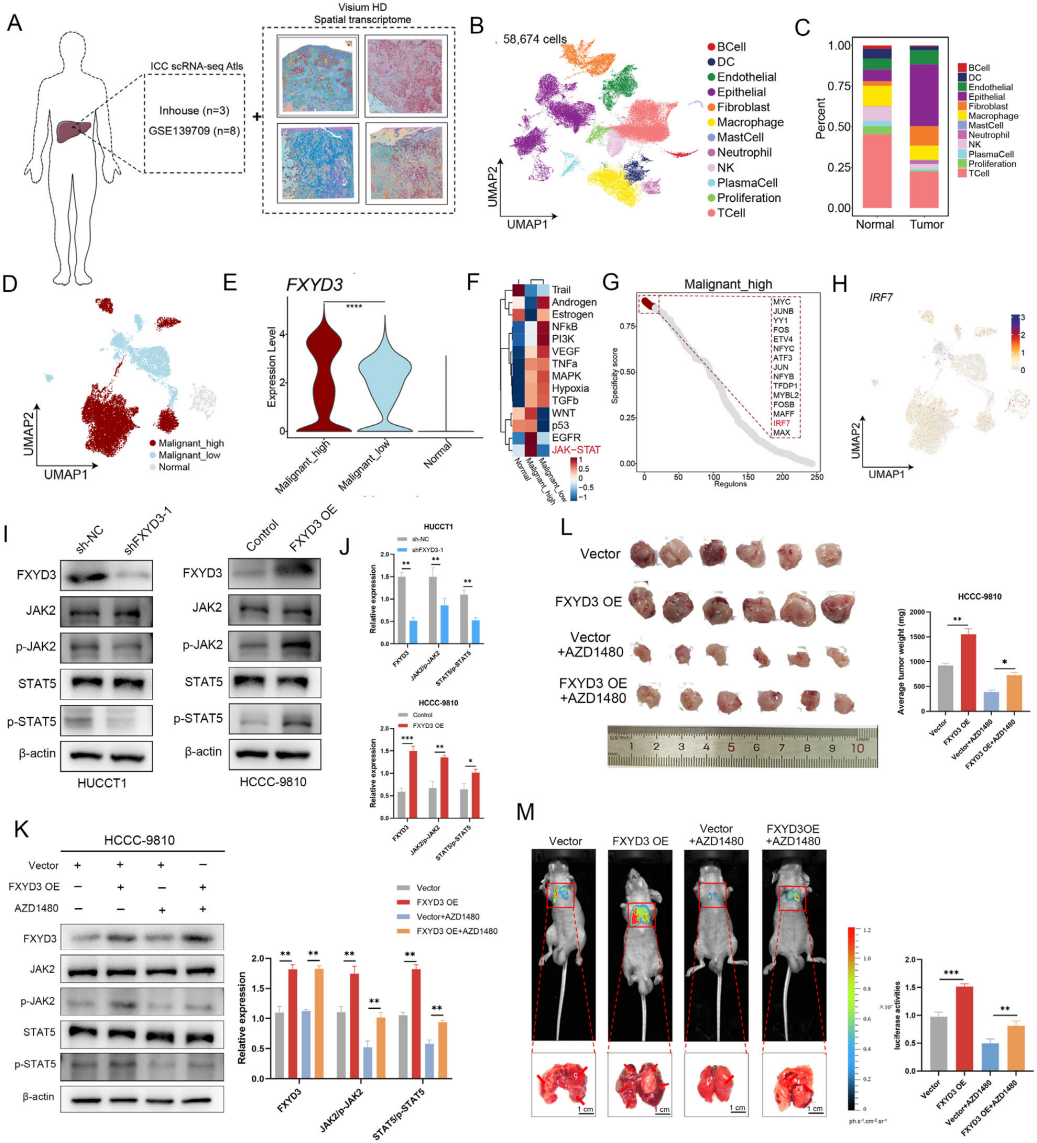
FXYD3 contributes to the malignant progression of ICC by promoting JAK2/STAT5 signaling. A) Integration of single‐cell transcriptomic data with high‐resolution spatial transcriptomics for comprehensive analysis. B) Uniform manifold approximation and projection (UMAP) plots showing the cell type distribution. Dots represent individual cells, and colors represent differential cell types. C) Bar plots showing cell type enrichment differences in tumor and normal sample types. Colors represent different cell types. D) UMAP plots showing the malignant cell types. Dots represent individual cells, and colors represent differential malignant types. E) Violin plot showing the expression of FXYD3 in malignant cell types. F) Differential activation of PROGENy pathways. G) Top 15 ranked TFs in each malignant type. H) UMAP plots showing the expression and transcriptional activity of IRF7. I,J) Western blot analysis was performed to examine the expression of key molecules, including FXYD3 and the JAK2/STAT5 pathway. K) The expression levels of several core components of the JAK2/STAT5 signaling pathway were assessed by western blot analysis when HUCCT1 cells were treated with a JAK inhibitor (AZD1480). L) Representative images show ICC transplanted tumors in each group (*n* = 8) (Left); tumor weight are provided for each group (Right). M) Bioluminescence imaging images and representative gross images of lung metastases in each group (*n* = 8). Error bars show the mean ± SEM. ^*^ *p* < 0.05, ^**^ *p* < 0.01, and ^***^ *p* < 0.001. The *P* value was determined using two‐tailed unpaired Student's test or two‐way ANOVA. Data are representative of three independent experiments.

We integrated our in‐house and publicly available single‐cell datasets and performed a joint analysis with high‐resolution spatial transcriptome data obtained from the Visium HD platform (10x Genomics) to elucidate the underlying mechanism by which FXYD3 promotes ICC development (**Figure** **5**A). The integrated single‐cell dataset comprised 11 clinical samples encompassing 58674 single cells. Twelve major cell types were identified among the samples using classical lineage‐specific markers: B cells, dendritic cells, endothelial cells, epithelial cells, fibroblasts, macrophages, mast cells, neutrophils, natural killer (NK) cells, plasma cells, proliferating cells, and T cells (Figure 5B,C). Subsequently, we conducted a clustering analysis of epithelial cells to further investigate the transcriptomic signature and molecular heterogeneity of FXYD3‐high and FXYD3‐low malignant cells. Based on the origin tissue of the epithelial cells and their FXYD3 labeling scores, all epithelial cells were categorized into three distinct groups: an FXYD3‐high malignant group, an FXYD3‐low malignant group, and normal cells (Figure 5D). The analysis revealed that FXYD3 was markedly overexpressed in more aggressive tumor cells (Figure 5E). Additionally, FXYD3 substantially activated the JAK/STAT signaling pathway in malignant cholangiocytes (Figure 5F). Moreover, our findings indicated that IRF7 plays a pivotal role in the activation of the FXYD3 and JAK/STAT pathways (Figure 5G,H).

Furthermore, western blotting was performed to evaluate the expression levels of key proteins involved in the JAK2/STAT5 signaling pathway. The results demonstrated that the protein levels of phosphorylated JAK2 (p‐JAK2) and phosphorylated STAT5 (p‐STAT5) were significantly reduced in HUCCT1 cells with downregulated FXYD3 expression compared to their counterparts. Conversely, ICC‐9810 cells overexpressing FXYD3 exhibited the opposite trend in protein expression (Figure 5I,J). Subsequently, ICC‐9810 cells overexpressing FXYD3 were treated with 100 nmol L^−1^ of the JAK2‐specific inhibitor AZD1480 (AstraZeneca) for 48 h. Western blot analysis revealed that AZD1480 treatment markedly suppressed the upregulation of proteins involved in the JAK2/STAT5 signaling pathway (Figure 5K). Additionally, in vitro CCK‐8 and wound‐healing assays indicated that AZD1480 significantly inhibited the proliferation and migration of ICC‐9810 cells overexpressing FXYD3 compared to their counterparts (Figure S4A,B, Supporting Information). Consistent with these findings, AZD1480 substantially diminished the colony‐forming ability of ICC‐9810 cells overexpressing FXYD3 (Figure S4C, Supporting Information). EdU assay results further corroborated the inhibitory effect of AZD1480 on the proliferation of ICC‐9810 cells overexpressing FXYD3 (Figure S4D, Supporting Information). Next, we developed a subcutaneous tumor xenograft mouse model. The tumor growth rate was significantly inhibited while the average tumor weight and volume markedly decreased following the treatment of FXYD3‐overexpressing HCCC‐9810 cells with AZD1480 (Figure 5L; Figure S4E, Supporting Information). The TUNEL assay and Ki‐67 staining further confirmed that AZD1480 treatment attenuated the tumorigenic potential of HCCC‐9810 cells overexpressing FXYD3 (Figure S4F, Supporting Information). Additionally, luciferase‐labeled HCCC‐9810 cells overexpressing FXYD3 were treated with AZD1480 whereas BALB/c nude mice were intravenously injected for in vivo experiments. Compared to the corresponding control group, nude mice injected with AZD1480‐treated FXYD3‐overexpressing HCCC‐9810 cells exhibited significant suppression of lung metastasis (Figure 5M).

### 2.5 FXYD3 Exhibits a Direct Interaction With IRF7

**Figure 6:**
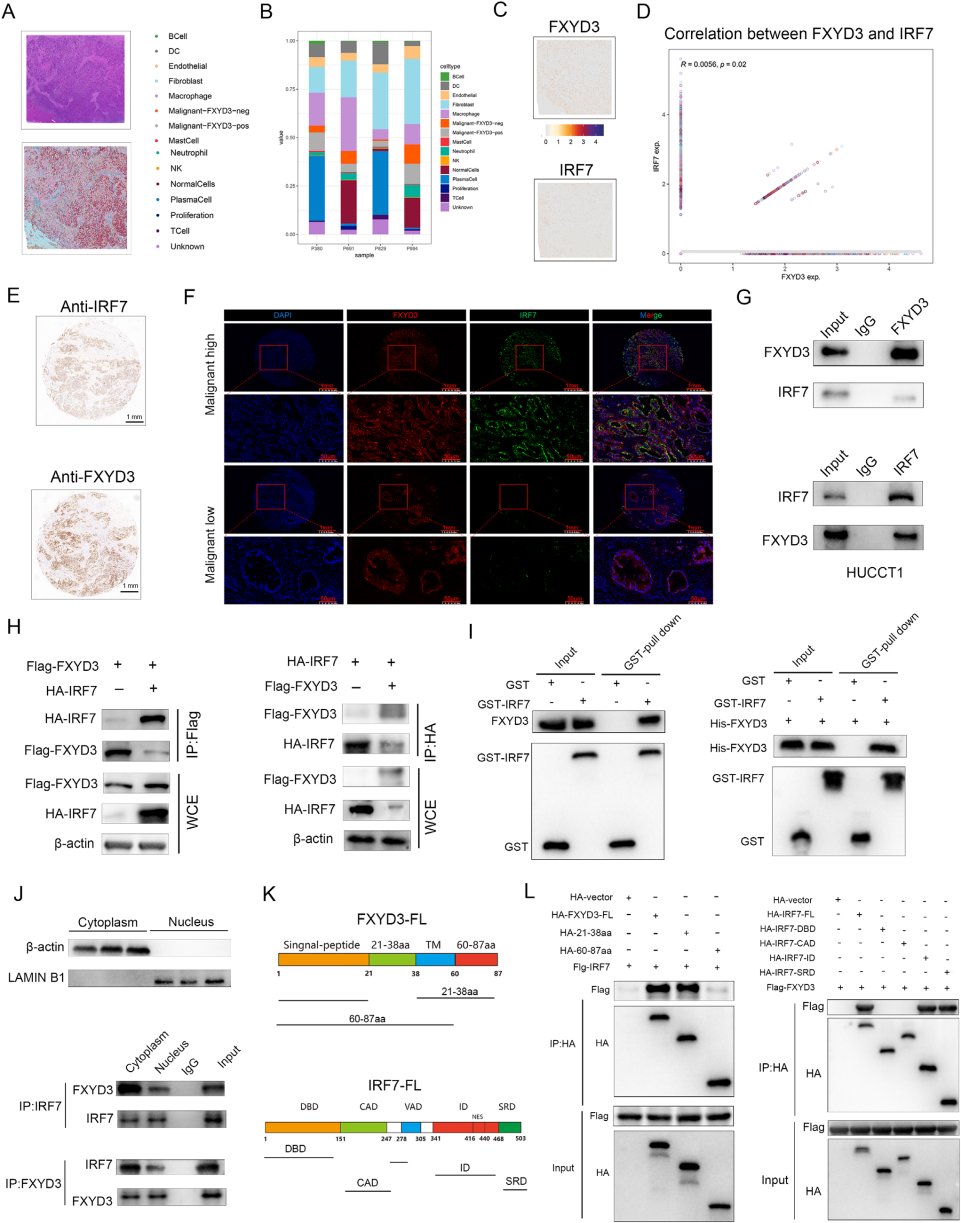
FXYD3 directly interacts with IRF7. A) The spatial distribution of FXYD3+ and FXYD3‐ malignant cells in four ICC samples was analyzed using the Visium HD platform (10x Genomics). B) The cell type composition of each sample. The samples exhibited heterogeneity in cellular composition. C,D) Spatial correlation of FXYD3 and IRF7 expression. E) Representative immunohistochemical images of anti‐FXYD3 and anti‐IRF7 staining (*n* = 30). F) Representative images of multiple immunofluorescence staining for FXYD3 and IRF7 (*n* = 20): the red box indicates FXYD3, the green box represents IRF7, and the blue box indicates DAPI staining. G) Co‐IP analyses were performed to examine the interaction between IRF7 and FXYD3 and between FXYD3 and IRF7 (bottom) in HUCCT1 cells. H) Left: Co‐IP analyses for HA‐IRF7 and Flag‐FXYD3 in HEK293T cells expressing Flag‐FXYD3 or Flag‐FXYD3 and HA‐IRF7. Western blotting was performed on WCEs (*n* = 2). Right: IP analyses for Flag‐FXYD3 and HA‐FXYD3 in HEK293T cells expressing HA‐IRF7 or Flag‐FXYD3 and HA‐IRF7. Western blotting was performed on WCEs (*n* = 2). I) Left: GST pull‐down assays with recombinant purified GST‐IRF7 and total proteins from HUCCT1 (*n* = 3). Right: GST pull‐down analysis of the interaction between His‐tagged FXYD3 and GST‐tagged IRF7 (*n* = 3). J) Up: The cytoplasmic and nuclear fractions of HUCCT1 cells were isolated using the Invent SC‐003 Minute Cytoplasmic and Nuclear Protein Extraction Kit, and the efficiency of separation was assessed by immunoblotting with antibodies against Lamin B1 (nuclear marker) and Actin (cytoplasmic marker). Down: Nuclear and cytoplasmic lysates were subjected to immunoprecipitation using antibodies against FXYD3, IRF7, or control rabbit IgG, respectively. The immunoprecipitated complexes were subsequently analyzed by immunoblotting with anti‐FXYD3 and anti‐IRF7 antibodies (*n* = 6). K,L) Co‐expression of HA‐vector, HA‐tagged FXYD3, HA‐21‐38aa mutant or HA‐60‐87aa mutant FLAG‐tagged IRF7 in HEK293T cells. Whole cell lysates were immunoprecipitated with anti‐HA antibody followed by immunoblotting using either anti‐HA or anti‐Flag antibodies; HA‐IRF7 and its deletion mutant were transfected into 293T cells together with Flag‐FXYD3. Whole cell lysates were then analyzed by western blotting using either anti‐HA or anti‐FLAG antibodies. Error bars show the mean ± SEM. ^*^ *p* < 0.05, ^**^ *p* < 0.01, and ^***^ *p* < 0.001. The *P* value was determined using two‐tailed unpaired Student's test or two‐way ANOVA. Data are representative of three independent experiments.

We further utilized the Visium HD platform (10x Genomics) to investigate the spatial distribution of FXYD3‐positive and FXYD3‐negative malignant cells in the four ICC samples (**Figure** **6**A). The cell‐type composition of each sample is presented in Figure 6B. Notably, the spatial correlation between FXYD3 and IRF7 expression underscores their colocalization within the spatially resolved samples (Figure 6C,D). Immunohistochemical analysis confirmed that the expression pattern of FXYD3 was consistent with that of IRF7 (Figure 6E). The relationship between FXYD3 and IRF7 was further clearly elucidated through multiple immunofluorescence techniques, demonstrating significant spatial co‐localization of the two proteins in high‐grade ICC samples compared to low‐grade ICC samples (Figure 6F). Next, we performed co‐immunoprecipitation assays, which confirmed the association between endogenous FXYD3 and IRF7, further validating the interaction between them (Figure 6G,H). Additionally, GST pull‐down assays revealed a direct physical interaction between the two proteins (Figure 6I).

Previous studies have demonstrated that the intracellular localization of IRF7 is closely associated with its functional status. In the resting state, IRF7 is predominantly localized to the cytoplasm, where it exists as an inactive monomer and does not engage in transcriptional regulation, thereby maintaining a poised state for activation. However, upon stimulation by specific signals, IRF7 undergoes phosphorylation, forms homo‐or heterodimers, and translocates from the cytoplasm to the nucleus. This stimulus‐induced shift in subcellular localization represents a critical regulatory mechanism underlying the transcriptional activity of IRF7. The cytoplasmic and nuclear fractions were isolated from HUCCT1 cells to determine the subcellular localization of the interaction between FXYD3 and IRF7. The purity of the fractions was validated using lamin B1 and actin as nuclear and cytoplasmic markers, respectively (Figure 6J, Top). Thereafter, immunoprecipitation assays were performed to examine the interaction between FXYD3 and IRF7 in each subcellular compartment. The results demonstrated that the interaction between FXYD3 and IRF7 predominantly occurred in the cytoplasmic fraction (Figure 6J**, Bottom**). Subsequently, a series of FXYD3 deletion mutants were engineered, and Co‐IP experiments were performed, which demonstrated that the HA‐60‐87aa domain of FXYD3 is critical for its interaction with IRF7 (Figure 6K **Up, L Left**). Moreover, various IRF7 deletion mutants were constructed to investigate the domains of IRF7 responsible for the interaction with FXYD3. The ID and SRD domains in IRF7 were found to be indispensable for maintaining its interaction with FXYD3 (Figure 6K**Down, L Right**).

### 2.6 FXYD3 Activates the JAK2/STAT5 Signaling Pathway by Binding to IRF7

**Figure 7:**
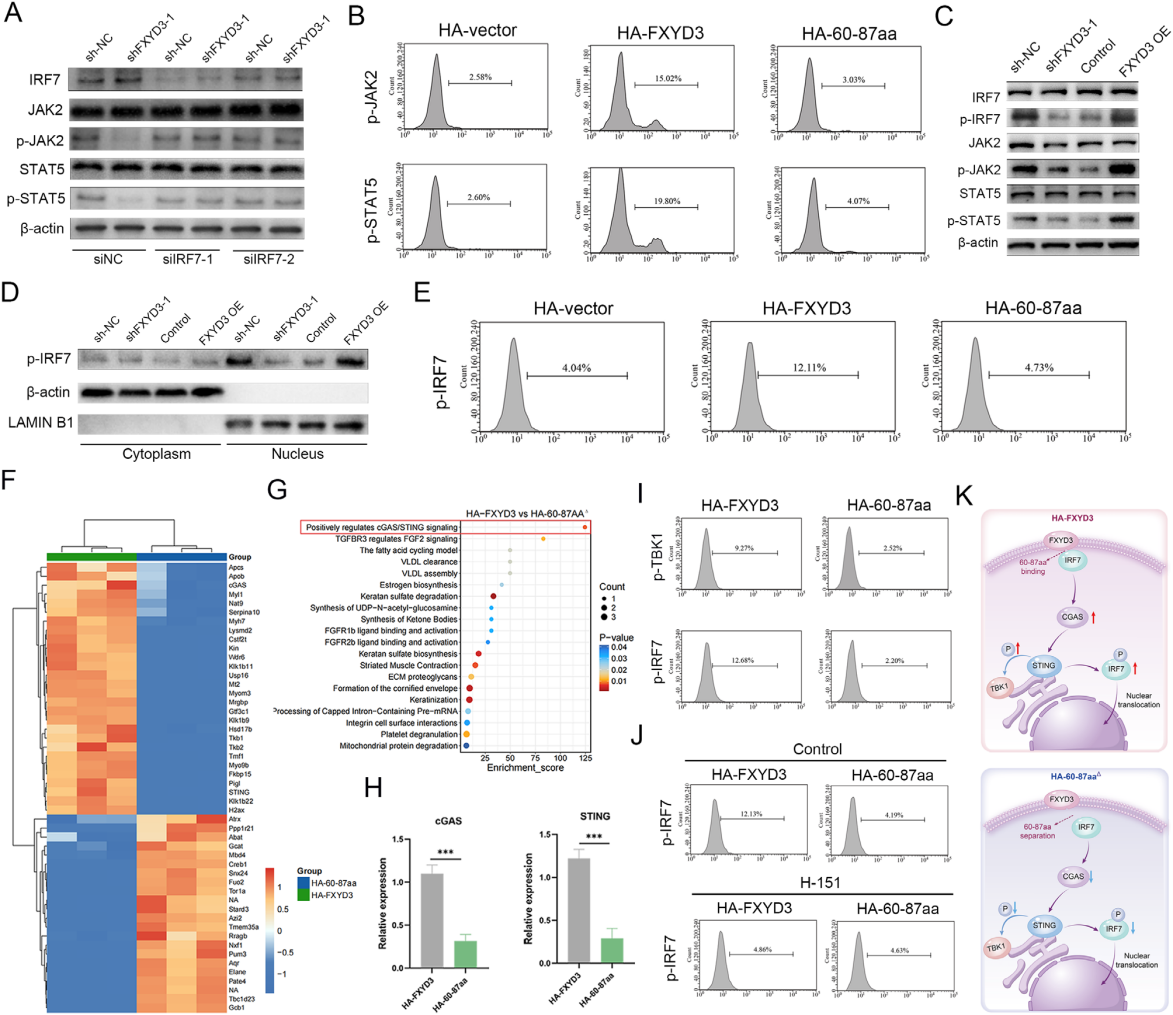
The FXYD3‐IRF7 interaction activates the cGAS/STING pathway to drive IRF7 phosphorylation. A) Western blot analysis was employed to examine the expression levels of core molecules involved in the JAK2/STAT5 pathway following knockdown of FXYD3 in HUCCT1 cells transfected with siRNA negative control (siNC) or siRNA targeting IRF7. B) The phosphorylation levels of core molecules of the JAK2/STAT5 pathway were detected by flow cytometry after transfection with HA‐vector, HA‐FXYD3, and its deletion mutant (HA‐60‐87aa). C) Western blot analysis was used to evaluate IRF7 phosphorylation and key protein expression in the JAK2/STAT5 pathway in HUCCT1 cells after FXYD3 knockdown, FXYD3‐overexpressing HCCC‐9810 cells, and their corresponding controls. D) Western blot analysis assessed IRF7 phosphorylation in different cellular fractions. E) Flow cytometry was employed to assess the impact of deleting the 60–87 domain of FXYD3 on IRF7 phosphorylation. F) The heat map shows the top 50 differentially expressed genes in different groups (*n* = 3). Color scale: blue denotes low expression, red denotes high expression. The blue module represents the HA‐60‐87aa deletion group, and the green module represents the full‐length HA‐FXYD3 group. G) Functional enrichment analysis of the full‐length HA‐FXYD3 group versus HA‐60‐87aa deletion group. H) qPCR was used to detect the expression levels of cGAS and STING in the full‐length HA‐FXYD3 group or the 60–87aa deletion group. I) The expression levels of p‐TBK and p‐IRF7 in the full‐length HA‐FXYD3 group or the 60–87aa deletion group were detected by flow cytometry. J) After treatment with the specific small molecule inhibitor of cGAS/STING H‐151, the phosphorylation level of IRF7 in the full‐length HA‐FXYD3 group and the 60–87aa deletion group was detected by flow cytometry. K) Graphical Abstract: the interaction between FXYD3 and IRF7 within the 60–87aa region activates the cGAS/STING pathway. This activation promotes the transition of IRF7 from an inactive state to a phosphorylated active form (p‐IRF7). Error bars show the mean ± SEM. ^*^ *p* < 0.05, ^**^ *p* < 0.01, and ^***^ *p* < 0.001. The *P* value was determined using two‐tailed unpaired Student's test or two‐way ANOVA. Data are representative of three independent experiments.

We transfected control small interfering RNA (siNC) or IRF7‐specific siRNA (siIRF7) into HUCCT1 cells with FXYD3 knocked down to further investigate whether FXYD3 regulates the JAK2/STAT5 signaling pathway by interacting with IRF7. The results demonstrated that transfection with siNC led to a reduction in the expression of p‐JAK2 and p‐STAT5 in FXYD3‐knockdown HUCCT1 cells. Conversely, the depletion of FXYD3 did not alter the phosphorylation status of p‐JAK2 and p‐STAT5 following transfection with IRF7‐specific siRNA (**Figure** **7**A). The relationship between FXYD3 and IRF7 was confirmed in vitro and in vivo. The results of the colony formation assay, EdU staining, scratch migration assay, and apoptosis detection indicated that compared with the control group cells, the inhibition of FXYD3 expression did not significantly affect the proliferation, invasion, or migration abilities of HUCCT1 cells with downregulated FXYD3 expression and transfected with siIRF7, nor did it cause a significant decrease in the apoptosis rate (Figure S5B–E, Supporting Information). Subsequently, we established mouse models of lung metastasis and orthotopic tumor implantation. Our results demonstrated that FXYD3 knockdown failed to significantly suppress lung metastasis in siIRF7‐transfected mice (Figure S5H, Supporting Information). In line with these findings, in a subcutaneously transplanted tumor mouse model, injection of FXYD3‐knockdown cells transfected with siIRF7 did not lead to a statistically significant decrease in mean tumor weight (Figure S5F, Supporting Information). These conclusions were further corroborated and reinforced by TUNEL and Ki‐67 staining (Figure S5G, Supporting Information). Additionally, considering that the 60–87aa domain of FXYD3 is associated with IRF7 interaction, we hypothesized that deletion of this domain may impede JAK2/STAT5 signaling. Accordingly, HA‐vector, HA‐FXYD3, and HA‐60‐87aa were transfected into the cells and analysed using flow cytometry and western blot analysis. Consistent with our expectations, deletion of the HA‐60‐87aa domain in FXYD3 abolished its ability to enhance JAK2/STAT5 signaling (Figure 7B; Figure S5A, Supporting Information). Collectively, these findings indicated that FXYD3 modulates JAK2/STAT5 signaling via its interaction with IRF7.

### 2.7 The FXYD3‐IRF7 Interaction Activates the cGAS/STING Pathway to Drive IRF7 Phosphorylation

The transformation of IRF7 into its activated state, phosphorylated IRF7 (p‐IRF7), is a pivotal molecular event in the orchestration of the JAK/STAT signaling cascade, marking a decisive turning point in cellular signal transduction. We first measured the phosphorylation levels of IRF7 and the expression of core proteins in the JAK2/STAT5 pathway in FXYD3‐knockdown HUCCT1 cells, FXYD3‐overexpressing HCCC‐9810 cells, and their corresponding control cells. The results showed that FXYD3 knockdown significantly suppressed the phosphorylation of IRF7, JAK2, and STAT5, whereas FXYD3 overexpression enhanced their phosphorylation (Figure 7C). Subsequently, western blot analysis of different cellular fractions revealed that changes in FXYD3 expression primarily affected the nuclear levels of phosphorylated IRF7 (Figure 7D). Notably, the flow cytometry results demonstrated that deletion of the 60–87aa domain of FXYD3 abolished its ability to enhance IRF7 phosphorylation (Figure 7E).

Next, we performed whole‐transcriptome sequencing to assess the impact of the 60–87aa region on other molecules or signaling pathways to investigate how FXYD3 influences IRF7 phosphorylation. The sequencing results indicated that compared with HA‐60‐87aa, full‐length HA‐FXYD3 significantly activated the cGAS/STING signaling pathway (Figure 7F,G). The cGAS/STING pathway serves as an upstream signaling hub for IRF7 activation and plays a key role in cancer and inflammatory diseases. Thereafter, we examined the activation status of the cGAS/STING pathway and IRF7 phosphorylation after transfection with HA‐FXYD3 or HA‐60‐87aa to validate these findings. The results confirmed that relative to HA‐60‐87aa, HA‐FXYD3 markedly increased the levels of cGAS, STING, p‐TBK1, and p‐IRF7 (Figure 7H,I). We then treated cells with H‐151, a specific small‐molecule inhibitor of cGAS/STING, to further verify that IRF7 activation was mediated through the cGAS/STING pathway. The results showed that the inhibition of cGAS/STING signaling prevented the enhancement of IRF7 phosphorylation, even upon HA‐FXYD3 transfection (Figure 7J). In summary, our findings demonstrated that the interaction between FXYD3 and IRF7 within the 60–87aa region activates the cGAS/STING pathway. This activation promotes the transition of IRF7 from an inactive state to a phosphorylated active form (p‐IRF7), facilitating its dimerization and subsequent translocation from the cytoplasm to the nucleus (Figure 7K).

### 2.8 Type I Interferon Mediates the Interaction Between FXYD3‐IRF7 and JAK2/STAT5

**Figure 8:**
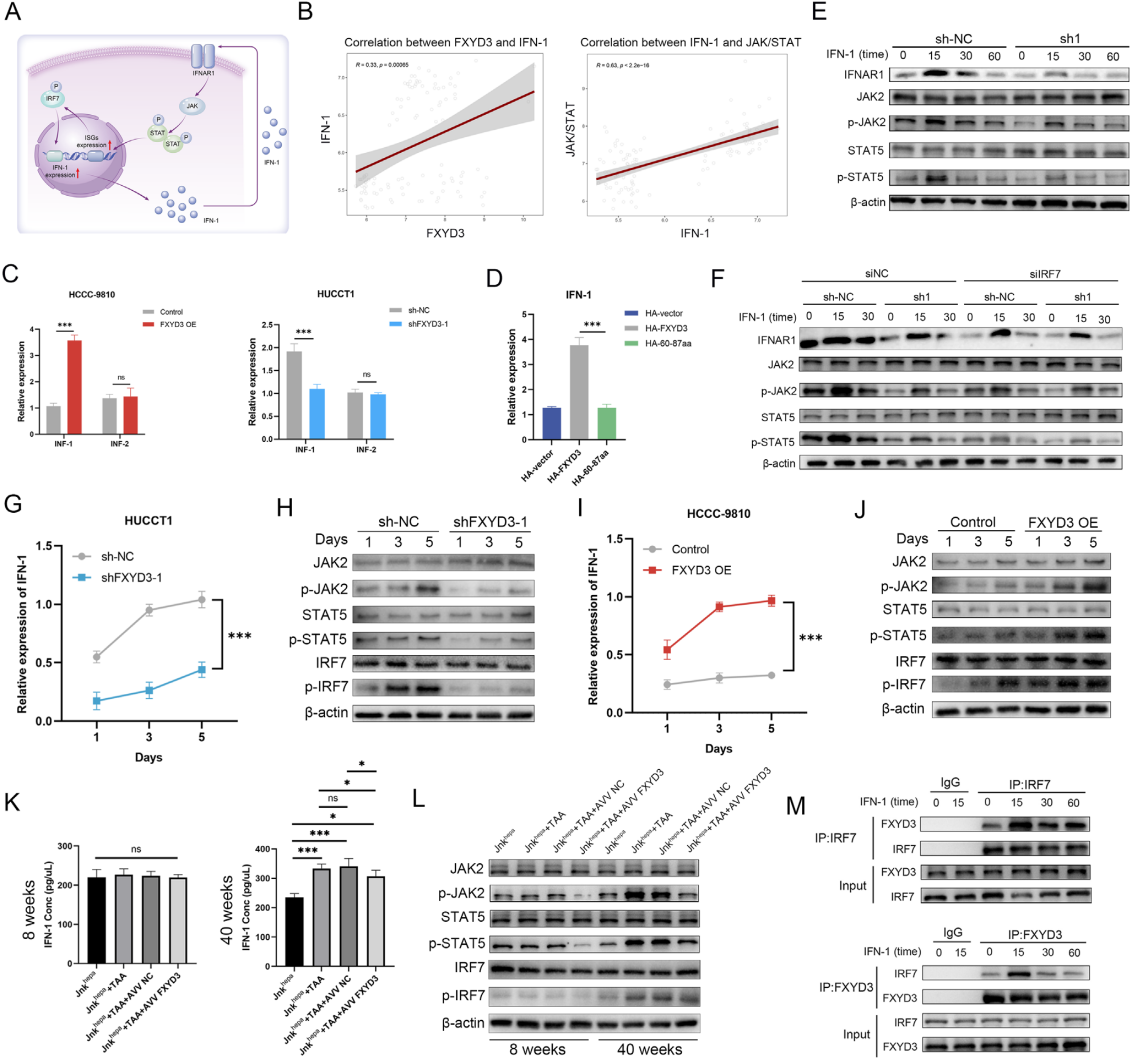
Type I interferon mediates the interaction between FXYD3‐IRF7 and JAK2/STAT5. A) Graphical Abstract: IRF7 phosphorylation and nuclear translocation initiate a signaling cascade wherein IFN‐I production activates the JAK/STAT pathway via IFNAR1, inducing ISGs and forming the IRF7‐IFN‐I positive feedback axis. B) Left: Correlation analysis between FXYD3 expression and IFN‐1 expression. Right: Correlation analysis between FN‐1 expression and JAK/STAT pathway. C) The expression levels of IFN‐1 and IFN‐2 were determined by qRT‐PCR in cells with FXYD3 knockdown or overexpression (*n* = 6). D) Following transfection of HA‐vector, HA‐FXYD3, or HA‐60‐87aa, the expression levels of IFN‐1 and IFN‐2 were assessed using qRT‐PCR (*n* = 6). E) Western blot analysis was performed to analyze the expression of core molecules in the JAK2/STAT5 pathway in HUCCT1 cells with FXYD3 knockdown after stimulation with IFN‐1 (50 ng mL^−1^). F) HUCCT1 cells with FXYD3 knocked down were transfected with si‐NC or siIRF7 and stimulated with IFN‐1 for detection of core molecule expression in the JAK2/STAT5 pathway using western blot. G–J) qPCR and western blot analysis were used to measure the expression levels of IFN‐I, the core components of the JAK2/STAT5 signaling pathway, and the phosphorylation level of IRF7 on days 1, 3, and 5 in FXYD3‐knockdown HUCCT1 cells, FXYD3‐overexpressing HCCC‐9810 cells, and their corresponding control cells. K,L) The expression levels of IFN‐I, the core components of the JAK2/STAT5 signaling pathway, and the phosphorylation level of IRF7 were detected by ELISA or western blot at 8 and 40 weeks in a spontaneous ICC animal model. M) Protein immunoprecipitation was performed using antibodies against FXYD3 or IRF7 in IFN‐treated cells for subsequent immunoblot analysis. Error bars show the mean ± SEM. ^*^ *p* < 0.05, ^**^ *p* < 0.01, and ^***^ *p* < 0.001. The *P* value was determined using two‐tailed unpaired Student's test or two‐way ANOVA. Data are representative of three independent experiments.

IRF7 functions downstream of PRR signaling and is a master regulator of IFN‐I production. Following phosphorylation and nuclear translocation, IRF7 drives the transcription of IFN‐I genes. Secreted IFN‐I then binds to the IFNAR on the cell surface, activating the JAK/STAT signaling pathway, which culminates in the expression of ISGs, forming a positive feedback loop termed the IRF7‐IFN‐I axis (**Figure** **8**A). Our previous findings indicated that FXYD3 enhances JAK2/STAT5 signaling through its interaction with IRF7, which led to the hypothesis that the binding of FXYD3 to IRF7 promotes interferon secretion, subsequently triggering JAK2/STAT5 signaling. Accordingly, we first examined the correlation between FXYD3 and IFN‐I expression as well as the association between IFN‐I and JAK/STAT signaling pathway activity at the mRNA level in HUCCT1 cells. The results revealed significant correlations between FXYD3 and IFN‐I and IFN‐I and the JAK/STAT signaling pathway (Figure 8B). Next, we assessed the mRNA levels of IFN‐1 and type II interferon (IFN‐2) in HUCCT1 cells with FXYD3 knocked down and in HCCC‐9810 cells overexpressing FXYD3, respectively. The results indicated a positive relationship between the expression levels of IFN‐1 and FXYD3, whereas no notable alteration was observed in the expression levels of IFN‐2 following FXYD3 knockdown or overexpression (Figure 8C). Subsequently, we assessed IFN‐1 expression following various splicer treatments (HA vector, HA‐FXYD3, and HA‐60‐87aa) to further elucidate the correlation between IFN‐1 expression and the FXYD3‐IRF7 complex. The results also showed a considerable reduction in IFN‐1 expression when the binding domain of FXYD3 to IRF7 was disrupted (Figure 8D). Furthermore, FXYD3‐knockdown HUCCT1 cells and their control cells were treated with IFN‐1 for 5, 15, 30, and 60 min. Western blotting results demonstrated that IFN‐1 partially restored the reduced expression of p‐JAK2 and p‐STAT5 caused by FXYD3 knockdown (Figure 8E). Thereafter, HUCCT1 cells with FXYD3 knocked down, transfected with siNC or siIRF7, were stimulated with IFN‐1. The phosphorylation of p‐JAK2 and p‐STAT5 induced by IFN‐1 was substantially attenuated in siIRF7 cells compared to that in siNC cells (Figure 8F).

We then conducted a systematic series of time‐course experiments to evaluate the dynamic changes in key molecular markers. Specifically, we assessed the expression levels of IFN‐I, the core component of the JAK2/STAT5 signaling pathway, and the phosphorylation status of IRF7 in FXYD3‐knockdown HUCCT1 cells, FXYD3‐overexpressing HCCC‐9810 cells, and their corresponding control cells on days 1, 3, and 5. The results demonstrated that FXYD3 knockdown consistently suppressed the upregulation of IFN‐I expression and the phosphorylation of JAK2, STAT5, and IRF7 over time (Figure 8G,H). Conversely, FXYD3 overexpression led to a significant enhancement in IFN‐I expression, increased levels of key proteins in the JAK2/STAT5 pathway, and elevated IRF7 phosphorylation (Figure 8I,J). Next, we measured the expression levels of IFN‐I and the phosphorylation level of IRF7 in a spontaneous ICC animal model at 8 and 40 weeks. Consistent with the in vitro findings, compared to control mice, ICC mice with liver‐specific FXYD3 knockdown exhibited significantly reduced IFN‐I secretion and decreased phosphorylation levels of JAK2, STAT5, and IRF7 (Figure 8K,L). Notably, our co‐IP experiments confirmed that IFN‐1 stimulation augmented the interaction between FXYD3 and IRF7 (Figure 8M), indicating a positive feedback relationship between them. Finally, the IFNAR1 receptor blocker anifrolumab was used to validate the rescue experiment. The results demonstrated that following anifrolumab treatment of HUCCT1 cells, FXYD3 knockdown no longer significantly reduced the expression levels of core proteins in the JAK2/STAT5 signaling pathway and suppressed the interaction between FXYD3 and IRF7 (Figure S6A,B, Supporting Information). Collectively, these findings suggest that the interaction between FXYD3‐IRF7 and JAK2/STAT5 is mediated by IFN‐I.

### 2.9 Targeting FXYD3 Inhibits Tumor Progression and Increases Sensitivity to Gemcitabine and Cisplatin Combination Therapy

**Figure 9:**
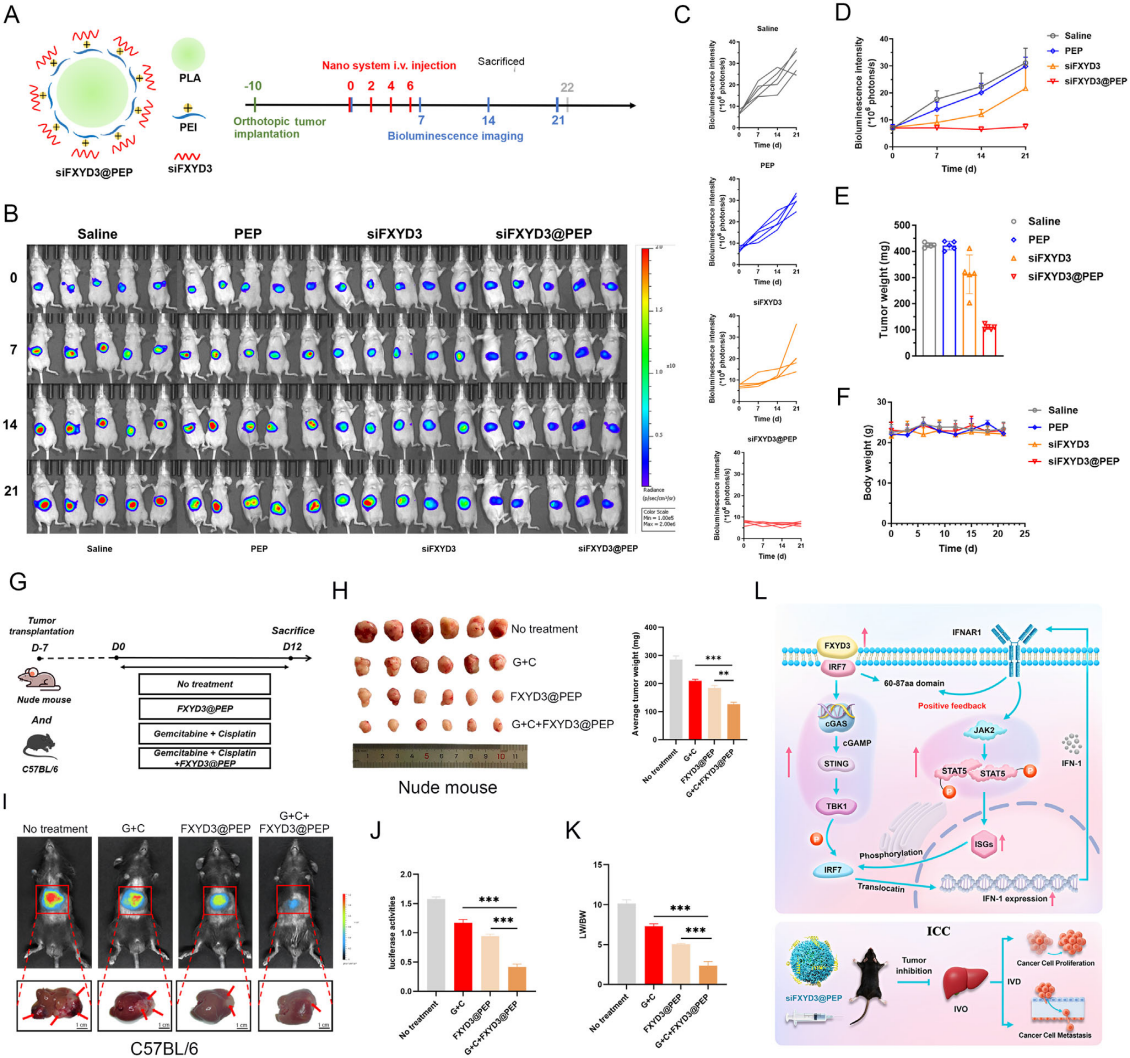
Nanoparticle‐encapsulated siRNA targeting FXYD3 inhibited the development of ICC. A) Structure of the siFXYD3@PEP nanodelivery system and treatment protocol. B–D) Representative images of bioluminescence imaging of ICC orthotopic transplanted tumor models under different treatments and grouping at different time points, fluorescence statistical map. E, F) Tumor and body weight of mice (*n* = 5). G) Experimental design of treatment with FXYD3@pep, either as a monotherapy or in combination with gemcitabine and cisplatin, in nude mice and spontaneous tumor mouse models. H) Left: Representative images of ICC‐transplanted tumors in each group (*n* = 6). Right: Statistical analysis of tumor weights in each group. I) Bioluminescence imaging and representative macroscopic images of liver tumors for each group (*n* = 8). J) Statistically analyzed bioluminescence imaging intensity in each group. K) Liver weight/body weight (LW/BW). L) Graphical Abstract: FXYD3 is a key oncogenic factor in intrahepatic cholangiocarcinoma progression and promotes tumor development by enhancing JAK2/STAT5 signaling through IRF7 binding. Nanoparticle‐encapsulated siRNAs targeting FXYD3 reduced tumor growth in a mouse model.

An siFXYD3 nano‐delivery system (siFXYD3@PEP) for treating an orthotopic xenograft tumor model of ICC was developed to investigate the possibility of FXYD3 as a therapeutic target for anticancer treatment (**Figure** **9**A, left). Nude mice were inoculated with orthotopic tumor cells (HUCCT1‐luc) followed by siFXYD3@PEP administration on days 0, 2, 4, 6 (*n* = 5) (Figure 9A, right). The TEM image in Figure S7A (Supporting Information) illustrates the morphology of siFXYD3@PEP particles, which were ≈150 nm in size and had a zeta potential of +15 mV. Cy5‐tagged siFXYD3 was incorporated into the delivery system to confirm successful loading of siFXYD3 onto siFXYD3@PEP and visualized using a three‐dimensional structured illumination microscope to capture its fluorescence signal (Figure S7B,C, Supporting Information). Pearson's correlation test revealed that cy5‐labeled siFXYD3 exhibited a strong correlation with Psc@DPP nanoparticles (Pearson correlation coefficient = 0.85) (Figure S7C, Supporting Information).

We established an orthotopic xenograft animal model of ICC to investigate the in vivo anti‐tumor effects of siFXYD3@PEP. First, HUCCT1 tumor cells were subcutaneously implanted into 4‐week‐old male BALB/c mice. Ten days later, the mice were randomly divided into four groups of five mice each. The siFXYD3@PEP nanoparticles were intravenously injected every two days, four times in total (on days 0, 2, 4, and 6) and bioluminescence imaging was performed weekly (on days 0, 7, 14, and 21). Our results demonstrated that saline or PEP injection did not impede tumor growth; however, siFXYD3 injection modestly inhibited tumor survival. Notably, mice treated with siFXYD3@PEP displayed significantly enhanced anti‐tumor efficacy compared to the other groups (Figure 9B–D). On the last day of the experiment (day 22), all mice were euthanized, and the tumors were removed and weighed. The tumors from the siFXYD3@PEP‐treated mice were substantially smaller than those from the other groups (Figure 9E). Notably, this decrease in tumor weight was not attributed to significant toxicity, as all groups showed similar increases in body weight during the treatment period (Figure 9F). Subsequently, a comprehensive assessment was conducted to examine the targeting specificity, in vivo stability, safety profile, biodistribution in non‐tumor organs, and potential off‐target effects of siFXYD3@PEP. Compared to free siFXYD3, siFXYD3@PEP demonstrated enhanced tumor tissue targeting and prolonged circulation time in vivo (Figure S8A,B, Supporting Information). Furthermore, comprehensive hematological analyses using an automated hematology analyzer indicated the favorable biocompatibility of siFXYD3@PEP (Figure S8C,D, Supporting Information). Additional histopathological evaluation using H&E and immunohistochemical staining revealed no significant adverse effects on major non‐tumor organs, including the heart, spleen, lungs, and kidneys (Figure S8E,F, Supporting Information).

Given that FXYD3 demonstrated potent anti‐tumor activity in vitro and in vivo, we hypothesized that combining FXYD3@PEP with first‐line chemotherapy would yield enhanced therapeutic efficacy. Accordingly, gemcitabine in combination with cisplatin was selected as the standard first‐line regimen, and combination intervention experiments were conducted in two animal models to assess the synergistic anti‐tumor effects (Figure 9G). The results demonstrated that the combination of FXYD3@PEP with gemcitabine and cisplatin exerted a more pronounced antitumor effect than FXYD3@PEP alone or gemcitabine and cisplatin combination therapy in nude mice and spontaneous tumor formation mouse models (Figure 9H–K).

Taken together, our findings demonstrated that FXYD3 directly interacted with IRF7 via its 60–87aa domain, thereby initiating a positive feedback loop mediated by the cGAS/STING pathway, and is amplified by IFN‐I. This loop leads to sustained activation of the JAK2/STAT5 signaling pathway, which ultimately drives the malignant progression of ICC (Figure 9L).

## 3 Discussion

ICC is a rare and understudied biliary malignancy with a dismal prognosis. Most patients miss the opportunity for radical surgery upon symptom onset.^[^ ^22^ ^]^ Moreover, the efficacies of chemotherapy, radiotherapy, and immunotherapy are limited for patients diagnosed with unresectable ICC.^[^ ^23^, ^24^ ^]^ Additionally, the precise pathogenesis of ICC remains unclear. Therefore, further exploration of the molecular mechanisms underlying ICC is imperative to improve patient survival outcomes. FXYD3 belongs to the FXYD domain‐containing Na^+^/K^+^ ATPase regulatory factor family and is highly expressed in various malignant tumors, including breast, prostate, and pancreatic cancer.^[^ ^25^, ^26^, ^27^, ^28^ ^]^ Although this protein is associated with unfavorable prognostic factors, such as invasion, migration, and lymph node involvement,^[^ ^29^ ^]^ its role in ICC progression remains unclear. We integrated single‐cell RNA sequencing, spatial transcriptomics, and functional genomics, which systematically revealed the role of FXYD3 as a key oncogenic driver in ICC and its underlying molecular mechanisms. Notably, FXYD3 directly interacts with IRF7 via its 60–87aa domain, thereby initiating a positive feedback loop mediated by the cGAS/STING pathway, and is amplified by IFN‐I. This loop leads to sustained activation of the JAK2/STAT5 signaling pathway, which ultimately drives the malignant progression of ICC. This finding positions FXYD3, a protein traditionally associated with ion channel regulation, at the crossroads of cancer‐related inflammation and innate immune signaling, thereby providing a new paradigm for understanding the pathogenesis of ICC.

IRF7 acts as a major regulator of IFN‐1 production and enhances IFN‐I transcription via phosphorylation and nuclear translocation. The secreted IFN‐I binds to IFNAR on the cell surface, leading to activation of the JAK/STAT signaling pathway. This cascade subsequently induces ISG expression, thereby establishing a positive feedback loop, known as the IRF7‐IFN‐I axis.^[^ ^30^, ^31^ ^]^ The most significant breakthrough of this study was the discovery of a direct physical interaction between FXYD3 and IRF7. Our data demonstrated that by binding to the ID and SRD domains of IRF7, FXYD3 effectively hijacked the activation process. This interaction occurs primarily in the cytoplasm and depends on the 60–87aa domain of FXYD3. This finding is of considerable significance because it reveals that FXYD3 directly modulates a core component of innate immune signaling beyond its traditional role in ion transport regulation. Subsequently, Co‐IP, GST pull‐down, and domain deletion experiments confirmed the direct nature of this interaction and precisely mapped the key binding regions, thereby laying a structural foundation for the future development of inhibitors targeting this interaction. Next, we sought to delineate the upstream signaling mechanisms. Whole‐transcriptome sequencing and subsequent functional validation revealed that the formation of the FXYD3‐IRF7 complex activates the cGAS/STING‐TBK1 axis. This suggests that FXYD3 may act as a non‐canonical activator of the innate immune pathway, potentially by mimicking or promoting the production or sensing of endogenous ligands (such as cytosolic DNA) that trigger cGAS. The indispensable role of the cGAS/STING pathway in FXYD3‐mediated IRF7 phosphorylation was then confirmed using the specific inhibitor, H‐151. These findings provide a novel molecular explanation for the “constitutive” activation of immune signaling observed in certain tumors.

The JAK‐STAT pathway is a highly conserved signaling pathway that is important for various physiological processes, including hematopoiesis, differentiation, metabolism, and immune regulation.^[^ ^30^ ^]^ This pathway is activated by cytokines, interferons, growth factors, and specific molecules that drive the progression of multiple cancers such as melanoma, glioblastoma, lung, pancreatic, breast, and rectal cancers, cholangiocarcinoma, and prostate cancer.^[^ ^32^, ^33^, ^34^, ^35^, ^36^, ^37^, ^38^ ^]^ The “dual positive feedback loop” model established in this study represents the core mechanism by which FXYD3 potently drives the malignant progression of ICC. In the first loop (FXYD3–IRF7–IFN‐I axis), the FXYD3–IRF7–cGAS/STING signaling axis induces the phosphorylation and nuclear translocation of IRF7, initiating the transcription of IFN‐I. The secreted IFN‐I binds to its receptor IFNAR in an autocrine/paracrine manner, which in turn enhances the interaction between FXYD3 and IRF7 through a mechanism that we have experimentally validated. This self‐amplifying circuit ensures the sustained intensification of pro‐oncogenic signaling. In the second loop (IFN‐I‐JAK2/STAT5 axis), IFN‐I further promotes cell proliferation, survival, and the formation of an immunosuppressive microenvironment via activation of the canonical JAK2–STAT5 pathway. Notably, in vitro and in vivo experiments demonstrated that IRF7 knockdown or treatment with the IFNAR1‐blocking antibody anifrolumab effectively disrupted these loops and reversed the oncogenic effects of FXYD3, confirming the functional necessity of this signaling axis.

Thus, the results of our study have a clear translational clinical value. First, FXYD3 is enriched in highly malignant ICC cell clusters, and its high expression is significantly associated with large duct‐type ICC, advanced pathological stage, and poor patient prognosis. Moreover, FXYD3 demonstrates superior diagnostic performance compared to CA19‐9, positioning it as a promising diagnostic biomarker and an independent prognostic indicator. Notably, the targeted FXYD3 nano‐delivery system (siFXYD3@PEP) exhibited significant antitumor efficacy in spontaneous and transplanted tumor models and markedly enhanced the sensitivity of ICC to standard gemcitabine and cisplatin chemotherapy. These findings suggest that targeting FXYD3 may be a potential therapeutic strategy, which may also overcome chemoresistance, offering a new therapeutic avenue for patients with advanced ICC.

However, the precise upstream trigger for the FXYD3–IRF7 interaction that activates the cGAS/STING pathway and whether it is linked to genomic instability or to mitochondrial stress remains unclear. Furthermore, the mechanism by which FXYD3‐driven IFN‐I response precisely modulates the function of immune cells—such as T cells and NK cells—within the tumor microenvironment requires additional investigation.

## 4 Conclusion

Our study systematically demonstrated for the first time that FXYD3 directly binds to IRF7, ingeniously hijacking and amplifying innate immune signaling to form a robust, self‐sustaining, dual positive feedback loop that drives the malignant progression of ICC. These findings enhance our understanding of ICC pathogenesis and reveal a novel cross‐talk mechanism between inflammation and tumorigenesis in cancer as well as establish FXYD3 as a highly promising diagnostic marker, prognostic indicator, and therapeutic target, paving a new path for precision therapy in ICC.

## 5 Experimental Section

### Data Acquisition

Public scRNA‐Seq datasets, specifically, GSE138709, GSE151530, GSE171899, and GSE201425, datasets were obtained from the GEO database. Additionally, the HRA001748 dataset was obtained from the genome sequence archive. The expression profiles and clinical data of the patients with 83 tumor samples from the GSE89749 dataset were also collected and collated. Furthermore, the expression matrix and clinical information of 38 patients with iCCA and eight normal samples from the TCGA‐CHOL dataset were downloaded from the Xena platform. RNA‐seq data and clinical information of patients in the Fudan iCCA cohort (FU‐iCCA) from the Biosino NODE database (NODE database: OEP001105) were also accessed.

### Single‐Cell Transcriptome and Differential Expression Analysis

CellRanger (v6.0.2) was used for read mapping and gene expression quantification, while the Seurat package (v4.1.1) was used for downstream analysis. Cells with > 1000 UMIs or > 15% of mitochondrial genes were excluded. Subsequently, the scVI algorithm was used to remove the effects between samples in single‐cell data to cope with batch effects and prevent the domination of individual patient features. In addition, the 4000 most variable genes were selected using the FindVariableFeatures function with accordingly scaled data to reduce the dimensionality and identify cell subtypes. Principal Component Analysis (PCA) was then performed using the above‐mentioned genes. Thereafter, the nearest neighbors were determined for graph clustering in accordance with the top 50 principal components using the FindNeighbour function. The FindCluster function was then applied to obtain the resulting clusters, which were visualized using a uniform manifold approximation and projection (UMAP) algorithm. Differential gene expression analysis between clusters was performed using the “FindAllMarkers” function. Wilcoxon's rank‐sum test with the Benjamini–Hochberg method was used to obtain and adjust *p*‐values for the comparisons.

### Gene Set Level for scRNA‐Seq Data Analysis

The RunAUCell function in the Pochi R package (v0.1.0) was used for single‐cell signature scoring. The differential signature score enrichment between groups was determined using a dual‐sided Wilcoxon rank‐sum test featuring the Benjamini–Hochberg FDR correction. Weighted Correlation Network Analysis (WGCNA) was used to identify co‐expression patterns in extensive gene expression datasets. Subsequently, the scWGCNA package (https://github.com/CFeregrino/scWGCNA), a modification of the original WGCNA technique for single‐cell datasets, was used to establish a co‐expression network of the genes expressed in the integrated dataset.

### Pathway and Cytokine Signaling Signature Analysis

Pathway and cytokine signaling analyses were conducted separately on the epithelial clusters using PROGENy and CytoSig, respectively. The scores were computed using the run_aucell function from the decoupleR package (v2.2.2) using the PROGENy network model and Cytosig Python packages with standard parameters. The top 1000 target genes of the progeny model, as suggested for the single‐cell data, were used for further analysis.

### Function Enrichment and Transcription Factor Regulon Analysis

KEGG enrichment analysis was conducted on differentially expressed gene sets using the clusterProfiler R package (v4.7.1.2). Pathways with adjusted *P*‐values < 0.05 were considered significantly enriched by DEGs. PySCENIC was used to analyze the regulatory network and regulon activity, from which subgroup‐specific transcription factors (TF) were obtained using the Wilcoxon rank‐sum test. In addition, a regulon‐associated specific score (RSS) within a certain cell type was constructed based on the Jensen–Shannon divergence, which was calculated using the philentropy (v0.6.0) package.

### Patient Samples, Cell Lines, and Lentiviral Transfection

This study included 273 patients with ICC who underwent complete resection at Nanjing Drum Tower Hospital between 2017 and 2022, after excluding those treated with neoadjuvant chemotherapy or those diagnosed with distant metastasis. Before the experiments, a portion of the tissue sample was processed and stored in formaldehyde solution, whereas the remaining portion was frozen using liquid nitrogen. The clinicopathological information of the patients at diagnosis, including age, sex, pathological stage, microvascular invasion, maximum tumor diameter, histological type, tumor recurrence, and survival status, was obtained from a predesigned database. This study was approved by the Ethics Committee of the Drum Tower Clinical College of Nanjing University Medical School (2024‐989‐1) and was conducted in accordance with the Declaration of Helsinki and government policies. All the participants provided written informed consent.

Human normal bile duct cell lines (HIBEC, primary extraction) and cholangiocarcinoma cell lines (HUCCT1 RRID: CVCL_0324, HCCC‐9810 RRID: CVCL_6908, and RBE RRID: CVCL_4896) were obtained from the Cell Bank of the Shanghai Institute of Biological Sciences, Chinese Academy of Sciences. The cell lines were authenticated to ensure that they were free of contamination and subsequently cultured in Dulbecco's modified Eagle's medium (DMEM; Gibco) supplemented with 10% fetal bovine serum (FBS; Gibco) and 1% antibiotic solution (streptomycin/penicillin; Gibco). The cell lines were maintained at 37 °C in a humid environment, with 5% carbon dioxide, for up to 20 generations (2 months). A lentiviral vector encoding FXYD3 (LV‐FXYD3), a short hairpin RNA targeting FXYD3 (LV‐shFXYD3), and the corresponding empty vector (LV‐control/LV‐shNC) were purchased from GenePharma Biotech. Polyamines (Genepharma; 5 µg mL^−1^) were employed to enhance the infection efficiency. Stably transfected cell lines were selected by the addition of puromycin at a concentration of 6 µg mL^−1^ for seven days in succession.

### Western Blotting and Immunoprecipitation

Proteins were extracted from tissues and cell lines using RIPA buffer containing 1% protease inhibitor mixture (Cwbio, China) and 1% PMSF, followed by centrifugation at 14 000 g for 15 min. The proteins were then separated using sodium dodecyl sulfate‐polyacrylamide gel electrophoresis and loaded onto a polyvinylidene difluoride (PVDF) membrane (Millipore, USA). The membranes were blocked using a rapid sealant (Beyotime) after 30 min of lockdown followed by incubation overnight at 4 °C with specific primary antibodies, and horseradish peroxidase‐conjugated secondary antibody (1:2000, Abcam) at ambient temperature for 2 h. The samples were detected using enhanced chemiluminescence (ECL) reagents (Engreen, Beijing, China), and the optical densities of the protein bands were measured using an ECL western blot detection kit (Bio‐Rad).

In exogenous co‐IP experiments, epitope‐tagged plasmids were introduced into HEK293T cells, which were subsequently lysed in lysis buffer containing 5% glycerol, 1% Nonidet P‐40, 1 mm EDTA, 100 mm NaCl, 20 mm Tris‐HCl (pH 8.0), and protease inhibitors. The supernatant for each sample was incubated overnight at 4 °C with anti‐FLAG M2 beads (Sigma) or anti‐HA beads (MCE). Additionally, tissue samples were lysed and incubated with anti‐FXYD3 or anti‐IRF7 antibodies along with protein G magnetic beads (MCE). The antibodies used included FXYD3 (Abcam RRID: AB_2622552), JAK2 (Cell Signaling Technology RRID: AB_11207044), p‐JAK2 (Cell Signaling Technology RRID: AB_10805124), STAT5 (Cell Signaling Technology RRID: AB_11220013), p‐STAT5 (Cell Signaling Technology RRID: AB_656686), IRF7 (Abcam RRID: AB_854554), β‐actin (Cell Signaling Technology RRID: AB_2750915), anti‐rabbit IgG antibodies (Abcam RRID: AB_11212339), and anti‐mouse IgG antibodies (Abcam RRID: AB_482872).

### Cell Proliferation, Migration, and Invasion Assay Analysis

A Cell Counting Kit‐8 (CCK‐8; Vazyme Biotech Co., Ltd.) was used to assess the cell proliferation capacity according to the manufacturer's instructions. Briefly, 500 cells per well were seeded into 96‐well plates, followed by the addition of 10 µL CCK‐8 solution per well and incubation for 2 h. Absorbance was measured at 450 nm.

In the clone formation assay, cells were collected from each treatment group in the logarithmic growth phase, and cell suspensions were prepared. Subsequently, samples were generated using a gradient dilution method, inoculated into culture dishes with the medium, and cultured for 14 days. The process was terminated by visually detecting the clones in the containers. The supernatant was removed, and the samples were fixed with 4% paraformaldehyde. The cells were then stained with crystal violet solution for 30 min. Finally, the rate of clone generation was calculated.

A Transwell assay was performed using Transwell chambers (Millipore, USA) to assess cell migration and invasion. The upper chamber was seeded with the cell line and 200 µL DMEM medium without FBS, while the lower chamber was filled with 500 µL of complete medium containing 10% FBS to induce cell migration through the Transwell membrane. After incubation for one‐day, the medium was discarded and the Transwell chambers were washed twice with PBS. Subsequently, the cells were fixed using methanol for 20 min before staining the invasive and migratory cells with crystal violet (0.1%) at room temperature for half an hour in the dark. The stained cells were washed with PBS three times before examination under a light microscope.

In the wound healing assay, treated cells (density: 5×105 cells/well) were seeded into 6‐well plates. Upon reaching confluence, the cells were washed twice with PBS, and a uniform linear scratch was made in the center of the well with a sterilized pipette tip (volume: 200 µL). The distance of the wound edge was recorded at 0 h after two cycles of PBS rinsing. These results were used as the baseline. Images were collected from the same sites after 48 h, and the changes in distance were measured to investigate the extent of cell migration and repair.

### 5‐Ethynyl‐2‐Deoxyuridine (EdU) Assay

Cell proliferation capacity was evaluated using an EdU assay kit (RiboBio, Guangzhou, China), according to the manufacturer's instructions. Briefly, cell lines were seeded at a density of 2 × 104 cells/well in 96‐well plates and cultured in complete medium for 24 h. EdU (50 µmol/L; RiboBio) was added followed by incubation for 2 h at 37 °C. The cells were then fixed with 4% formaldehyde for 30 min and permeabilized with 0.5% TritonX‐100 for 10 min. The cells were washed thrice using PBS, and Edu‐positive cells were visualized using a combination of ApolloR reaction cocktail (1×400 µL) and Hoechest33342 (400 µL) under a microscope, and images were captured.

### Immunohistochemical (IHC) and Multiple Immunofluorescence Staining

The samples were fixed in 4% formaldehyde and embedded in paraffin. The paraffin blocks were sectioned into 4 µm slices, followed by incubation with primary antibodies against FXYD3 and ki‐67 (Abcam) at 4 °C for 12 h. Subsequently, the sections were incubated at room temperature with horseradish peroxidase‐labeled secondary antibodies for 1 h, followed by staining with 3,3′‐diaminobenzidine and hematoxylin. Two independent pathologists evaluated the staining positivity and intensity in a blinded manner. Ten random areas were selected for analysis from each slide.

Additionally, the tumor sections (5.0 µm thick) were examined using the multiplex immunofluorescence (mIF) technique. The sections were processed using an Opal fIHC kit (PerkinElmer, USA). The antibody staining procedure was applied to all sections, which were subsequently back‐stained with DAPI from Vector Laboratories, Germany. FXYD3 and IRF7 were detected using multiple immunofluorescence plates. All antibodies were diluted using an antibody diluent (Dako, Osaka, Japan). Secondary antibodies underwent treatment with ImmPRESSHRP (peroxidase) Polymer Cancer Detection Kit provided by Vector Laboratories, USA. The TSA reagents were diluted using 1 × Plus amplification diluent (PerkinElmer/Akoya Biosciences, 01752, USA).

### TUNEL Assay

TUNEL staining was used to detect DNA fragmentation features indicative of apoptosis in tumor sections embedded in paraffin and fixed with formalin. A Klenow‐Fragel DNA fragment assay kit (Roche, Basel, Switzerland) was used according to the manufacturer's instructions. Subsequently, ten random areas from each slice were selected for examination.

### Extraction of RNA and qRT‐PCR Assay

The Easypurure RNA kit (Reagent Bio) was used to separate RNA from samples according to the manufacturer's instructions. Briefly, tissues or cells were homogenized using lysis buffer, followed by the collection and centrifugation of lysates for 10 s. Subsequently, an equivalent volume of absolute ethanol was added, and the mixture was transferred to an RNA column. The mixture was centrifuged at 4000 g and 4 °C using a centrifuge tube. Thereafter, 500 µL of clean buffer was applied with 1 min of centrifugation, which then was repeated for another minute at 12 000 g in RNA‐free tubes. The RNA column was moved to a new RNase‐free tube and air‐dried for 2 min before treating with elution buffer (20 µL). After centrifugation 2 min at 12 000 g, the extracted RNA was preserved at −80 °C. The reaction mixture (20 µL/well) for qRT‐PCR studies contained total RNA (1 µg), HiScript IIqRT SuperMix (5×; Vazyme Biotechnology Co., LTD; 4 µL), and ddH2O. The ChamQTM Universal SYBR qPCR Master Mix system (Vazyme Biotech Co., Ltd) along with the following primer sequences were then used: FXYD3 forward sequence: 5′‐ GACGGATCCAACCTCTGCTCAGCCTGGT ‐3′ and reverse sequence: 5′‐ ATCGAATTCTCTTGCGAGAGGTGAGATGA ‐3.’

### Animal Experiments

All animal experiments were approved by the Animal Care and Use Committee of the Nanjing University Medical School (2023‐669‐1). Four‐week‐old female BALB/c nude mice were obtained from the Laboratory Animal Center of the Nanjing Medical University. Transfected cell lines (1×106 cells) were resuspended in 100 µL PBS and subcutaneously injected into nude mice (*n* = 6/group) and the growth of subcutaneous tumors was monitored. Additionally, cholangiocarcinoma cells expressing 1×106 luciferase (*n* = 12 per group) were injected into the tail vein to detect metastases. In‐situ and distant metastases were observed after five weeks using an IVIS100 imaging system (Caliper Life Sciences).

Subsequently, hepatocyte‐specific JNK1/2 knockout C57BL/6 mice (JNK∆hepa) and their associated ICC control models were provided by Professor Chaobo Chen, Xishan District People's Hospital, Wuxi City.

### Plasmid Construction and Transfection

FLAG‐tagged and HA‐tagged FXYD3, produced by PCR, were subcloned into the pcDNA3.1, eukaryotic expression vector (Invitrogen). Additionally, truncated FXYD3 was amplified from the HA full‐length FXYD3 expression vector and cloned into the pcDNA3.1‐HA expression vector. Expression plasmids for FLAG‐tagged IRF7 and HA‐tagged IRF7 or its variants (including IRF7‐DBD, IRF7‐CAD, IRF7‐ID, and IRF7‐SRD) were provided by Professor Chen Chaobo (Xishan District People's Hospital, Wuxi City, China). FXYD3 was cloned into the PGM‐puro lentiviral vector for overexpression. The plasmids were transfected into HEK293T cells using jetPRIME reagent (Polyplus) following standard protocols.

### RNA Interference Silencing

The small interfering RNA (siRNA) was transfected into cells using the Lipofectamine3000 transfection reagent (Invitrogen, L3000015) according to the manufacturer's instructions. The siRNA oligonucleotide sequences used were as follows: negative control siRNA (5′‐UUCUCCAAGCGUGACACUG‐3′), IRF7 siRNA (5′‐CAAGGUCUUAAUGAUGCGUG‐3′).

### Enzyme‐Linked Immunosorbent Assay

Whole blood was centrifuged at 3000 rpm and 4 °C for 5 min to isolate plasma. The IFN‐1 ELISA Kit (Elabscience) and IFN‐2 Kit for mouse plasma (Elabscience) were used according to the manufacturer's protocol. Optical density (OD) was assessed at the appropriate wavelength using a Synergy 2 multifunctional microplate detector (BioTek Instruments, Inc.).

### GST Pull‐Down Assay

Protein‐protein interactions between FXYD3 and IRF7 were analyzed using a glutathione S‐transferase (GST) pull‐down assay. Briefly, recombinant GST‐tagged IRF7 and His‐tagged FXYD3 were expressed in E. coli BL21(DE3) strains and purified using glutathione‐Sepharose beads (Cytiva) and Ni‐NTA agarose (Qiagen), respectively. Thereafter, ≈10 µg of purified GST or GST‐IRF7 protein was immobilized onto 20 µL of pre‐equilibrated glutathione‐Sepharose beads by incubating in binding buffer (50 mm Tris‐HCl, pH 7.5, 150 mm NaCl, 1 mm EDTA, 1% Triton X‐100, 1 mm DTT, and 1× protease inhibitor cocktail) at 4 °C for 2 h with gentle rotation. The beads were then washed three times with 1 mL of ice‐cold binding buffer to remove unbound proteins. Subsequently, an equimolar amount (or 10–15 µg) of purified FXYD3 was added to the GST or GST‐IRF7 bound beads in 500 µL of binding buffer. The mixture was incubated at 4 °C for 2–3 h with constant rotation to allow interaction. Beads were then pelleted by centrifugation at 1000 × g for 5 min at 4 °C and washed thoroughly with 1 mL of binding buffer five times to eliminate non‐specifically bound proteins. Finally, bound proteins were eluted by boiling the beads in 2× Laemmli sample buffer for 10 min, followed by separation by SDS‐PAGE and analysis by western blotting using anti‐FXYD3 and anti‐GST antibodies to confirm their interaction.

### Preparation, Characterization and Antitumor Therapy of siFXYD3@PEP

A siFXYD3 delivery nanosystem (siFXYD3@PEP) established using emulsification and solvent evaporation technology from Dr. Yihang Yuan was obtained. Briefly, a methoxy polyethylene glycol‐poly(lactic acid) copolymer (mPEG 5.5 kDa‐PLA 27 kDa, 10 mg) and polyethylene glycol‐poly(lactic acid) block copolymer (PEG 4.9 kDa‐PLA 27 kDa, 10 mg) were dissolved in dichloromethane in a 1:1 mass ratio to form a homogeneous organic phase. The organic phase was transferred to a round‐bottomed flask and emulsified with an aqueous phase containing the surfactant sodium cholate to form a stable emulsion. The emulsion was then stirred at 500 rpm for 24 h under magnetic stirring to allow the complete evaporation of dichloromethane and facilitate the self‐assembly of the nanoparticles. The resulting nanoparticles were collected by centrifugation at 15 000 rpm for 30 min, washed three times with deionized water to remove residual sodium cholate, and redispersed in double‐distilled water for storage.

Purified nanoparticles were modified with polyethyleneimine (PEI, 1.8 kDa in this study) via electrostatic adsorption to enhance the siRNA‐binding capacity. Briefly, the PEI 1.8 kDa solution was mixed with the nanoparticles at a 2:1 mass ratio and incubated at room temperature for 30 min to facilitate PEI adsorption onto the nanoparticle surface through electrostatic interactions. Unbound free PEI was removed by centrifugation (15 000 rpm for 15 min), followed by two washes with double distilled water. The PEI‐modified nanoparticles were then redispersed in double‐distilled water to obtain positively charged nanocarriers, which facilitated efficient siRNA loading. siFXYD3 was loaded onto PEI‐modified nanoparticles through electrostatic interactions. The PEI‐modified nanoparticles were mixed with siFXYD3 at a 30:1 mass ratio and incubated at room temperature for 30 min, allowing the positively charged PEI to attract the negatively charged siRNA for stable binding to the nanoparticle surface, thereby forming the final siFXYD3@PEP nanodelivery system. The complexes were collected by centrifugation at 14 000 rpm for 7 min to remove the unbound free siRNA, and the precipitate was resuspended in double‐distilled water for subsequent experiments.

An ICC orthotopic xenograft tumor model was established in female BALB/c nude mice. Ten days after the nude mice were inoculated with orthotopic tumor cells (HUCCT1‐luc), they were administered on days 0, 2, 4, and 6. Each group had 5 mice. Tumor burden was monitored weekly using bioluminescence imaging (IVIS spectral CT), and body weight was recorded throughout the study. The mice were euthanized on the final day (day 22) when the tumors were removed and weighed.

### Survival Analysis

Survival analysis was conducted using the R package survival. The Cox proportional hazards model was used to calculate the hazard ratios (HR) and 95% CIs. Additionally, the Survfit function was used to model the Kaplan–Meier survival curve. The Kaplan–Kaplan–Meier survival curves were compared using a two‐sided log‐rank test.

### Statistical Analysis

Quantitative data were analyzed using the independent samples *t*‐test or Mann–Whitney U test, where10as categorical variables were assessed using Pearson's chi‐squared (χ^2^) test. Paired samples were compared using paired *t*‐tests or Wilcoxon signed‐rank tests, while ranked data were analyzed using the Kruskal–Wallis H test or Mann–Whitney U test, depending on the context. Tumor volumes were evaluated using repeated‐measures analysis of variance (ANOVA). Survival curves were generated using the Kaplan–Meier method, and differences in survival rates were compared using the log‐rank test. All statistical analyses were performed using GraphPad Prism (version 9.2), SPSS (version 25.5), and R (version 4.2.0). Statistical significance was set at *p* < 0.05. 3. Data are presented as mean ± standard deviation (SD).

### Ethics Approval and Consent to Participate

This study was performed with the approval of the Research Ethics Committee of the Nanjing Drum Tower Hospital.

## Conflict of Interest

The authors declare no conflict of interest.

## Author Contributions

Y.Z., X.S., S.Z. contributed equally to this work. Y.Z. contributed to validation, methodology, data curation, and investigation and wrote the original draft. X.S. contributed to methodology and investigation. S.Z. contributed to validation and investigation. X.P. performed methodology. Y.L. acquired resources. X.H., A.Y. acquired resources and performed methodology. Y.Q. acquired resources and contributed to project administration. C.C., Y.Y. contributed to investigation and project administration. J.C. contributed to supervision, methodology, and project administration and wrote the original draft.

## Supporting information



Supporting Information

## Data Availability

Research data are not shared.
