# Supplementary material for: FXYD3 Promotes Tumor Progression by Binding With IRF7 to Regulate JAK2/STAT5 Signaling in Intrahepatic Cholangiocarcinoma
Source: Adv Sci (Weinh). 2025 Oct 30;13(3):e10782. doi: 10.1002/advs.202510782 (PMC12806521; doi:10.1002/advs.202510782)
Supplement: Supplementary file 1 — Supporting Information [file ADVS-13-e10782-s001.docx]

Supporting Information

**FXYD3 promotes tumor progression by binding IRF7 to regulate JAK2/STAT5 signaling in intrahepatic cholangiocarcinoma**

**
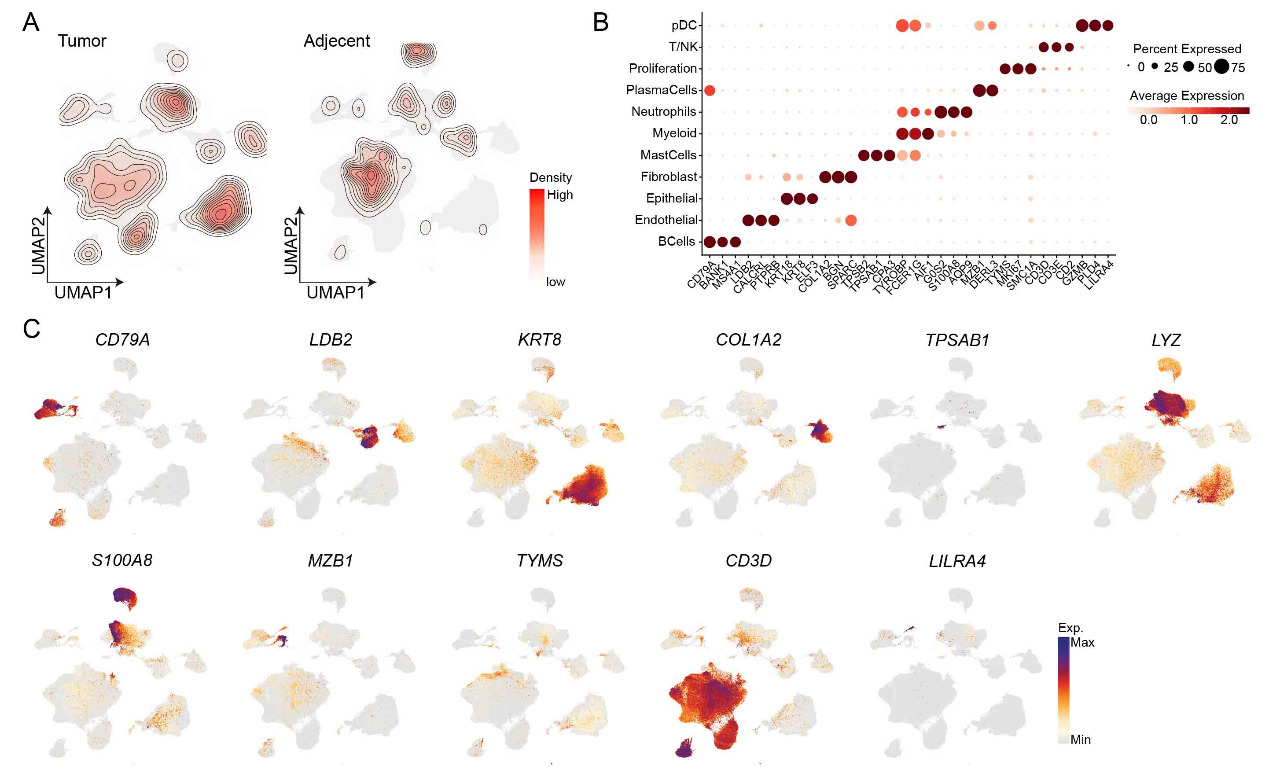
**

**Figure S1**. Identification of cell types in the integrated ICC scRNA-seq data. A. 2D density plots showing major cell population enrichment in tumor (left) and adjacent (right) samples. B. Dot plots showing the top 3 differentially expressed genes of each cell type. C. Uniform manifold approximation and projection (UMAP) plots showing the cell type markers. Dots represent individual cells, and colors from grey to red indicate the value from low to high.

**
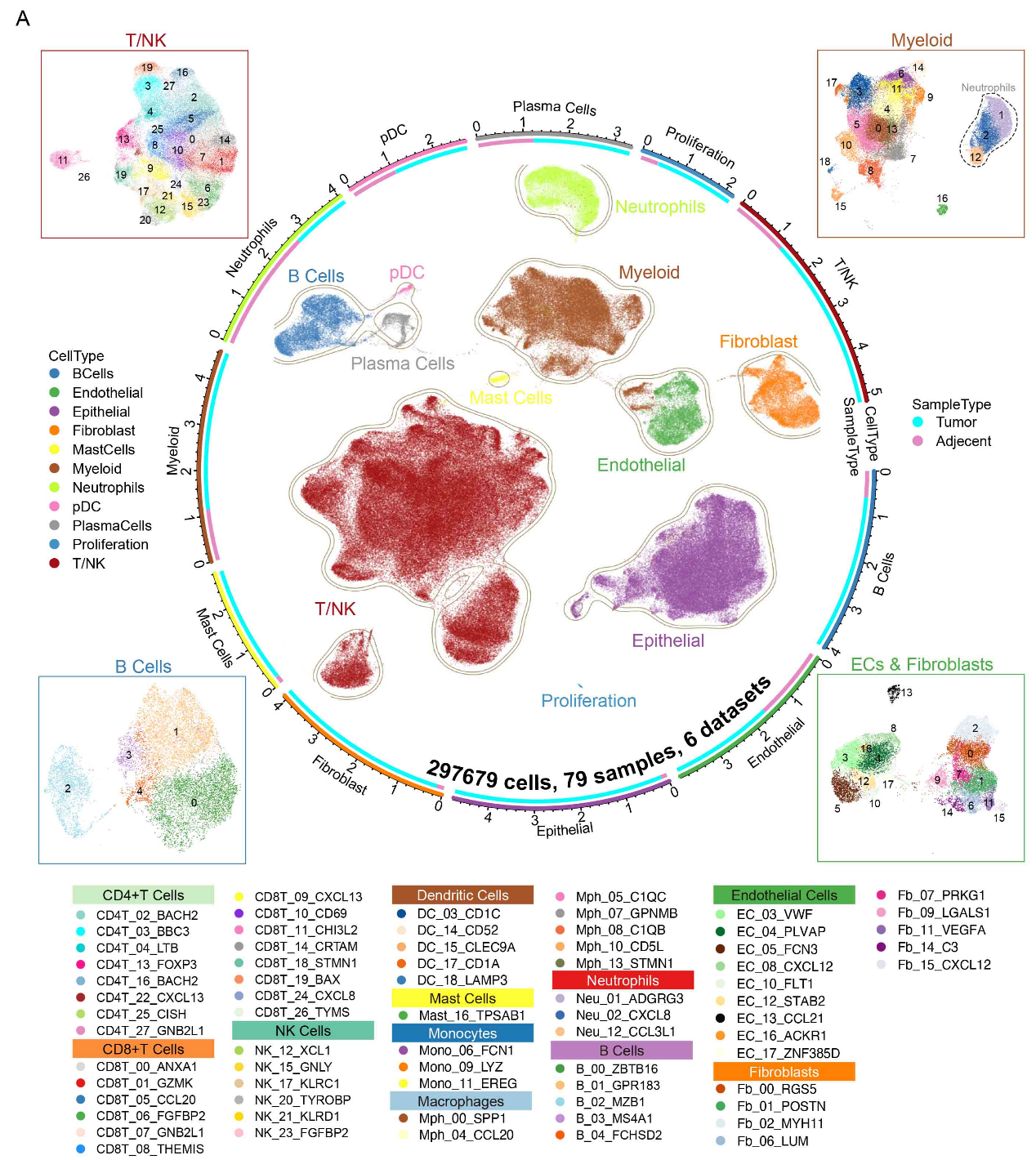
**

**Figure S2**. The ICC single-cell atlas. A total of 297, 679 high-quality cells from 79 samples are projected by Uniform manifold approximation and projection (UMAP) plot. Colors indicate the major cell types, and cluster boundaries are outlined by contour curves. The four corner insets show subclusters of T/NK cells, myeloid cells, B cells, and ECs & Fibroblasts. The axis outside the circular plot depicts the log scale of the total cell number for each cell class. The two-colored tracks (from outside to inside) indicate cell types and sample types.

**
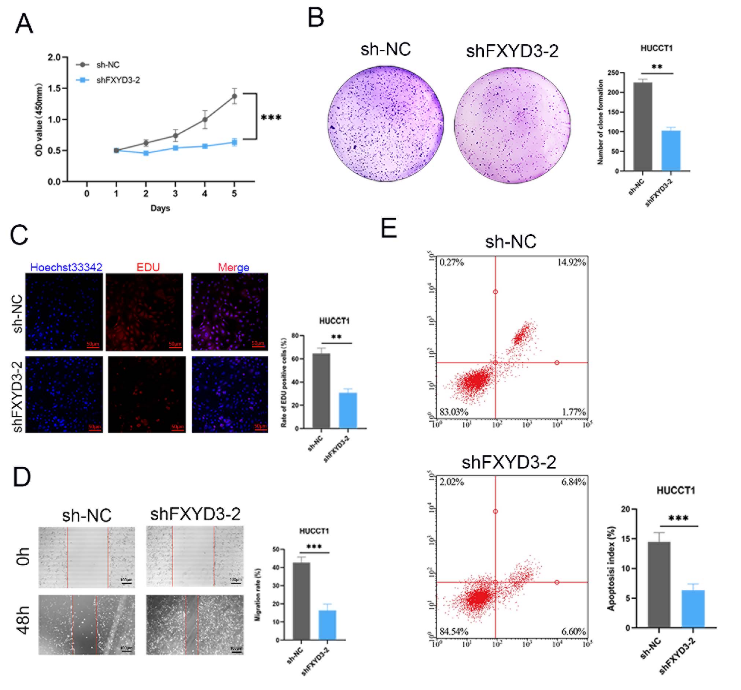
**

**Figure S3**. FXYD3 knockdown inhibits the malignant biological behavior of HUCCT1 cells. A. The proliferation ability of cells with FXYD3 knocked down was evaluated using the CCK-8 assay. B, C. Colony and EDU formation assays were utilized to assess the proliferation ability of cells with FXYD3 knockdown. D. The migration ability of cells with FXYD3 knocked down or overexpression was determined through the wound-healing assay. E. Apoptosis of ICC cell lines with FXYD3 knocked down analyzed by flow cytometry. Error bars show the mean ± SEM. *p < 0.05, **p < 0.01, and ***p < 0.001. The P value was determined using two-tailed unpaired Student’s test or two-way ANOVA. Data are representative of three independent experiments.

**
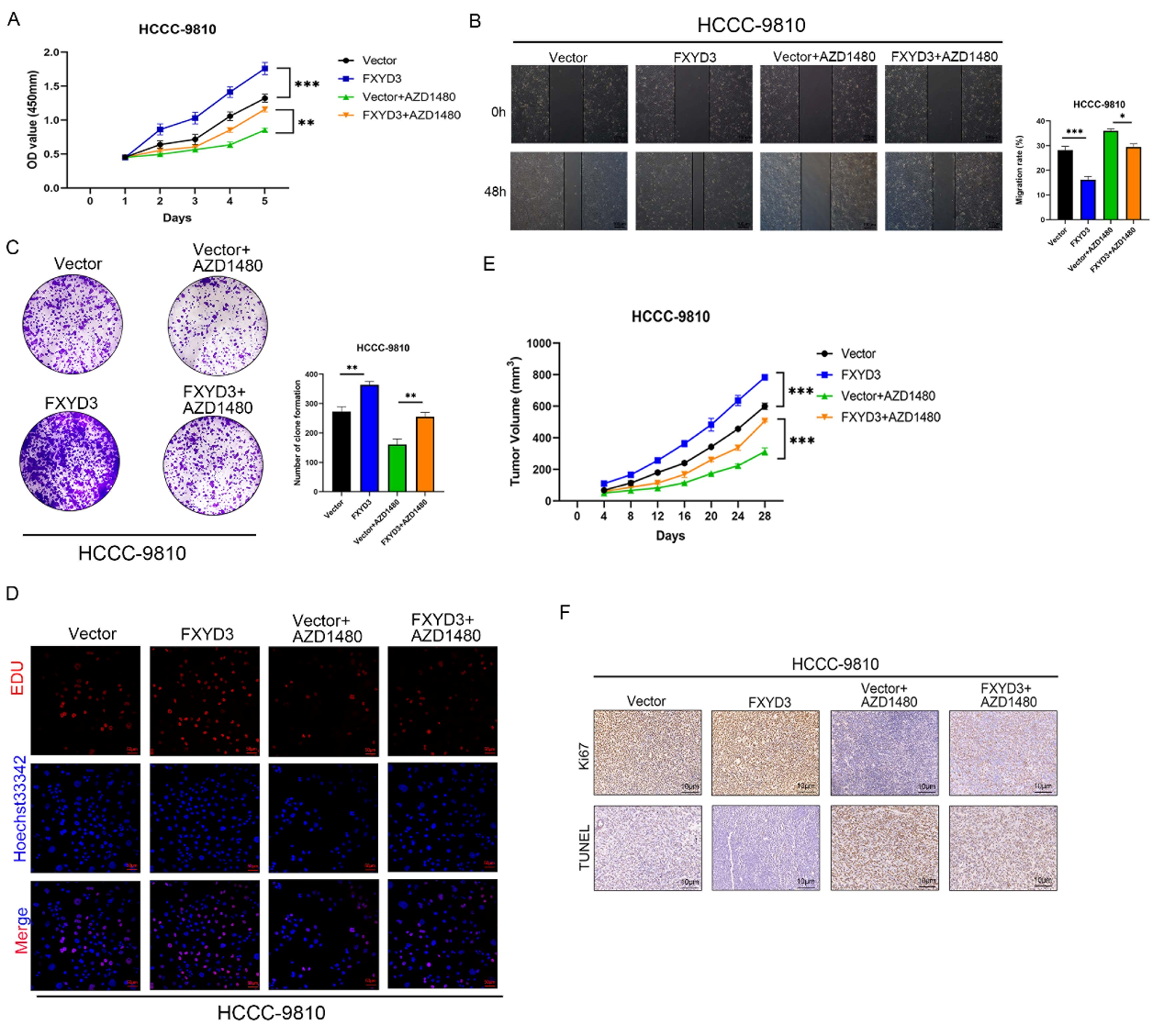
**

**Figure S4**. HUCCT1 cells overexpressing FXYD3 were treated with AZD1480. A, C, D. Cell proliferation was evaluated using CCK-8, colony formation, and EDU assays. B. Wound-healing assay was performed to assess the migration ability of HUCCT1 cells in each group. E. Tumor growth curves of each group. F. Immunohistochemical images depicting Ki-67 and TUNEL expression in transplanted tumors from each group. Error bars show the mean ± SEM. *p < 0.05, **p < 0.01, and ***p < 0.001. The P value was determined using two-tailed unpaired Student’s test or two-way ANOVA. Data are representative of three independent experiments.

**
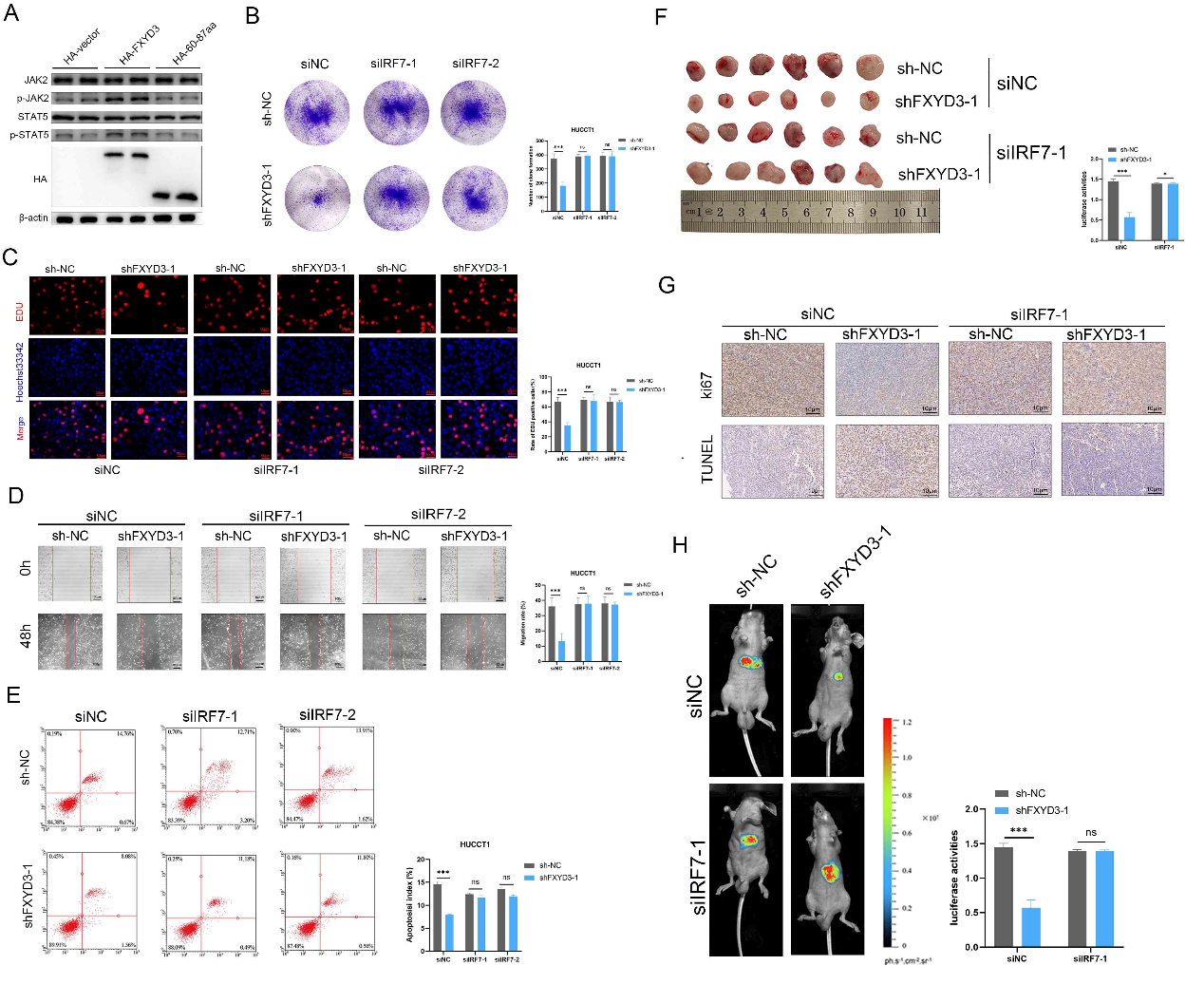
**

**Figure S5**. FXYD3 activates the JAK2/STAT5 signaling pathway by binding to IRF7. A. The expression levels of core molecules involved in the JAK2/STAT5 pathway were assessed by western blotting after transfection of HEK293T cells with either HA-vector, HA-FXYD3 or its deletion mutant (HA-60-87aa). B, C. Colony and EDU formation assays were utilized to assess the proliferation ability of cells HUCCT1 cells in each group. D. The migration ability of HUCCT1 cells in each group was detected by scratch assay. E. The proportion of apoptotic HUCCT1 cells was analyzed by flow cytometry. F. Typical images of ICC transplanted tumors in each group (n=8); tumor weight in each group. G. Representative immunohistochemical images of Ki-67 and TUNEL expression in each group of transplanted tumors. H. Representative images of bioluminescence imaging of orthotopic transplanted tumor models in each group (n=8). Error bars show the mean ± SEM. *p < 0.05, **p < 0.01, and ***p < 0.001. The P value was determined using two-tailed unpaired Student’s test or two-way ANOVA. Data are representative of three independent experiments.

**
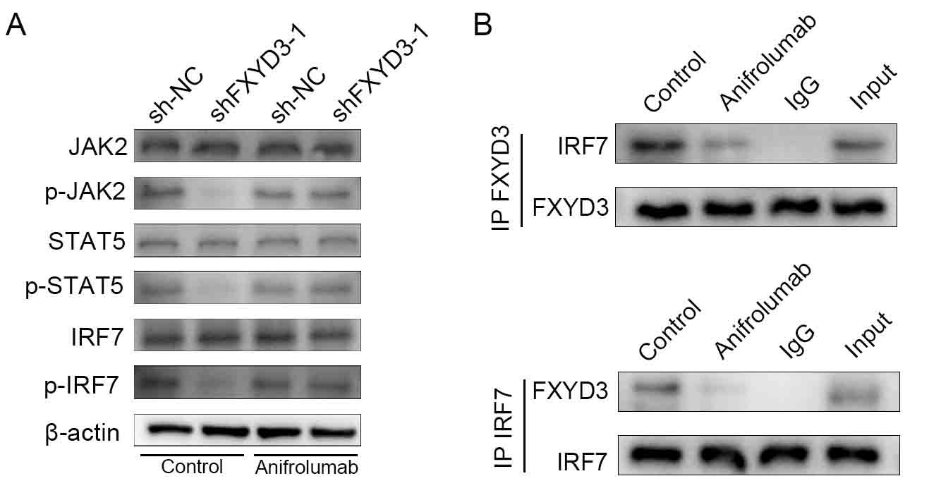
**

**Figure S6**. Anifrolumab inhibits FXYD3-induced activation of JAK2/STAT5 and IRF7 and their interaction. A. Treatment of HUCCT1 cells with the IFNAR1 blocker anifrolumab was followed by western blot analysis of core JAK2/STAT5 pathway protein expression and IRF7 phosphorylation. B. Immunoprecipitation was performed on cell lysates treated or untreated with anifrolumab using anti-FXYD3, anti-IRF7 antibodies or control rabbit IgG. The immunoprecipitation complexes were then analyzed by immunoblotting with anti-FXYD3 and anti-IRF7 antibodies (n = 3). Data are representative of three independent experiments.

**
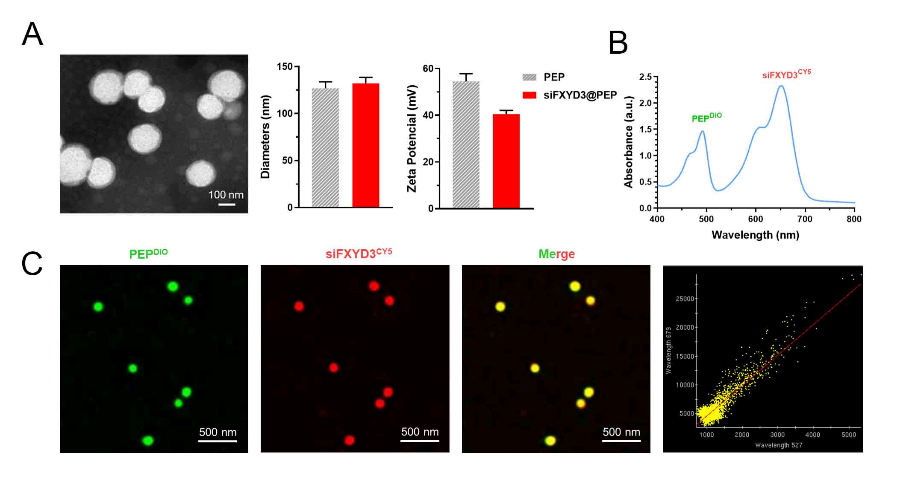
**

**Figure S7**. Nanorepresentation of siFXYD3@PEP. A. Transmission electron microscope image of siFXYD3@PEP, nanoparticle diameter, and zeta potential. B, C. The fluorescence signal corresponding to siFXYD3@PEP Cy5 on siFXYD3@PEP and Pearson correlation test. Data are representative of three independent experiments.

**
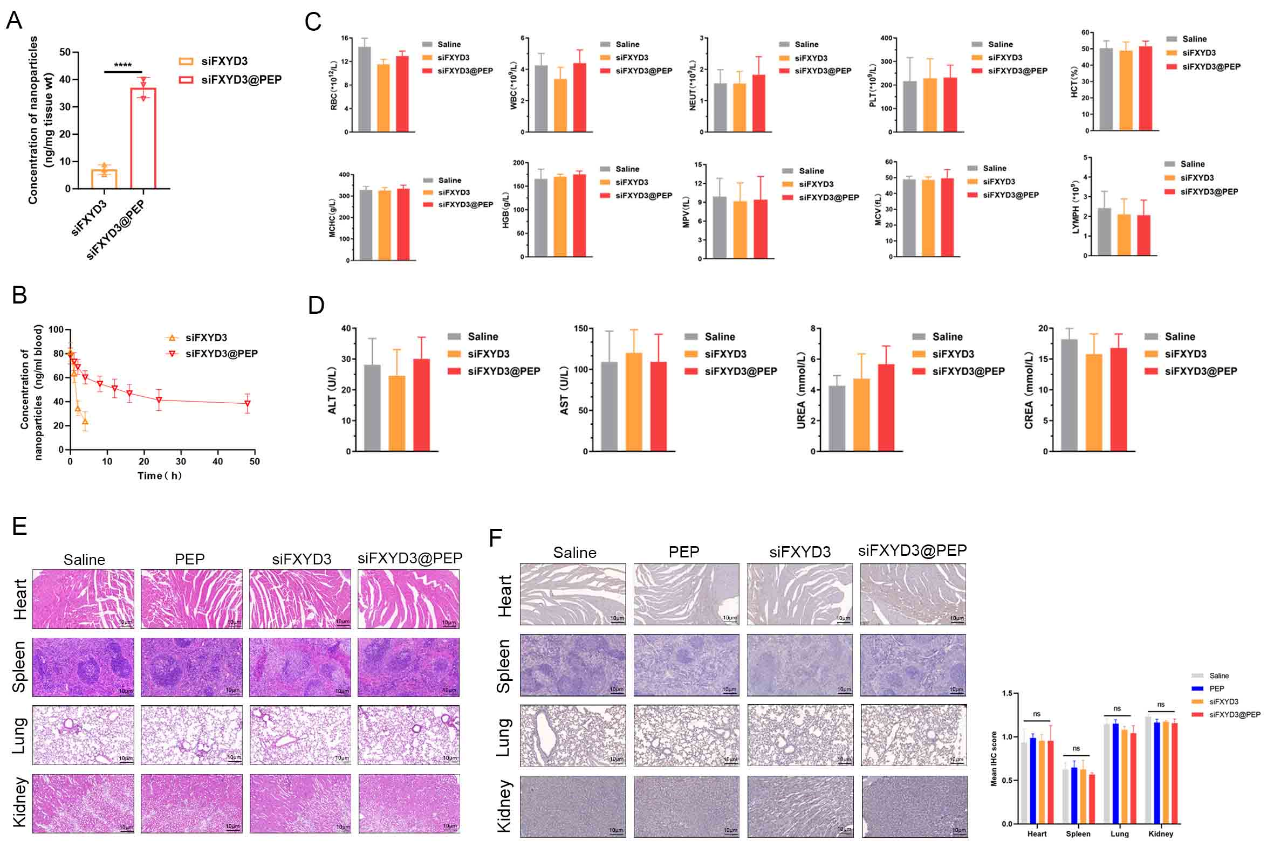
**

**Figure S8**. Biological safety evaluation of FXYD3@PEP. A. The content of siFXYD3 in tumor tissues was determined by the fluorescence calibration curve method. B. A time-dependent pharmacokinetic study was performed to evaluate the blood circulation stability of the siFXYD3@PEP nanosystem. C, D. The safety of the nanoparticle delivery system was assessed by complete blood count analysis and serum biochemical analysis. E, F. The effects of FXYD3@PEPE on major non-tumor organs were assessed by H&E staining and immunohistochemical staining. Error bars show the mean ± SEM. *p < 0.05, **p < 0.01, and ***p < 0.001. The P value was determined using two-tailed unpaired Student’s test or two-way ANOVA. Data are representative of three independent experiments.
